# The impact of taphonomic data on phylogenetic resolution: *Helenodora inopinata* (Carboniferous, Mazon Creek Lagerstätte) and the onychophoran stem lineage

**DOI:** 10.1186/s12862-016-0582-7

**Published:** 2016-01-22

**Authors:** Duncan J. E. Murdock, Sarah E. Gabbott, Mark A. Purnell

**Affiliations:** Department of Geology, University of Leicester, University Road, Leicester, LE1 7RH UK

**Keywords:** Panarthropoda, Lobopodia, Onychophora, Mazon Creek, Taphonomy

## Abstract

**Background:**

The origin of the body plan of modern velvet worms (Onychophora) lies in the extinct lobopodians of the Palaeozoic. *Helenodora inopinata*, from the Mazon Creek Lagerstätte of Illinois (Francis Creek Shale, Carbondale Formation, Middle Pennsylvanian), has been proposed as an intermediate between the “weird wonders” of the Cambrian seas and modern terrestrial predatory onychophorans. The type material of *H. inopinata*, however, leaves much of the crucial anatomy unknown.

**Results:**

Here we present a redescription of this taxon based on more complete material, including new details of the head and posterior portion of the trunk, informed by the results of experimental decay of extant onychophorans. *H. inopinata* is indeed best resolved as a stem-onychophoran, but lacks several key features of modern velvet worms including, crucially, those that would suggest a terrestrial mode of life.

**Conclusions:**

The presence of *H. inopinata* in the Carboniferous demonstrates the survival of a Cambrian marine morphotype, and a likely post-Carboniferous origin of crown-Onychophora. Our analysis also demonstrates that taphonomically informed tests of character interpretations have the potential to improve phylogenetic resolution.

**Electronic supplementary material:**

The online version of this article (doi:10.1186/s12862-016-0582-7) contains supplementary material, which is available to authorized users.

## Background

Lobopodians are extinct worm-like animals characterised by their unsegmented lobopodous limbs. Known principally from Lower Palaeozoic Lagerstätten, some lobopodian taxa have been proposed to have affinities with extant Onychophora, but their precise relationships are unclear; recent analyses have recovered lobopodian taxa as stem-Euarthropoda, stem-Panarthropoda and stem-Onychophora [1–4]. Correctly determining which clades particular lobopodian taxa are affiliated with has the potential to reveal important aspects of early panarthropod evolution. In the post-Palaeozoic fossil record, examples of unequivocal terrestrial onychophorans are known from Cretaceous [5] and Eocene [6, 7] amber. *Helenodora inopinata* Thompson and Jones [8], from the Mazon Creek Lagerstätte of Illinois (Francis Creek Shale, Carbondale Formation, Middle Pennsylvanian), is claimed as the oldest terrestrial lobopodian [8] and is generally regarded as a stem-onychophoran more closely related to extant onychophorans than to Cambrian lobopodians [9]. Yet the evidence for this phylogenetic placement is scant, with considerable uncertainty caused by poor preservation of details of the head. Some new details of the anterior region of *H. inopinata* have recently been described by Haug et al. [10], alongside a description of a second Mazon Creek lobopodian, *Carbotubulus waloszeki*. The discovery of additional specimens permits us to make a full redescription of *H. inopinata*, and to re-evaluate the interpretations of Thompson and Jones [8]. Furthermore, by incorporating the results of recent work on experimental decay of onychophorans into taphonomic analysis of character preservation we demonstrate that failure to include taphonomic evidence can lead to widespread loss of resolution in the results of phylogenetic analysis. Based on these new observations, in particular the absence of jaws, claws and slime papillae, we interpret *H. inopinata* as a stem-onychophoran and find no evidence for the proposed terrestrial mode of life.

## Results and discussion

### Systematic palaeontology

**Phylum ONYCHOPHORA** Grube 1853

**Genus HELENODORA** Thompson and Jones 1980

#### Remarks

**Fig. 1:**
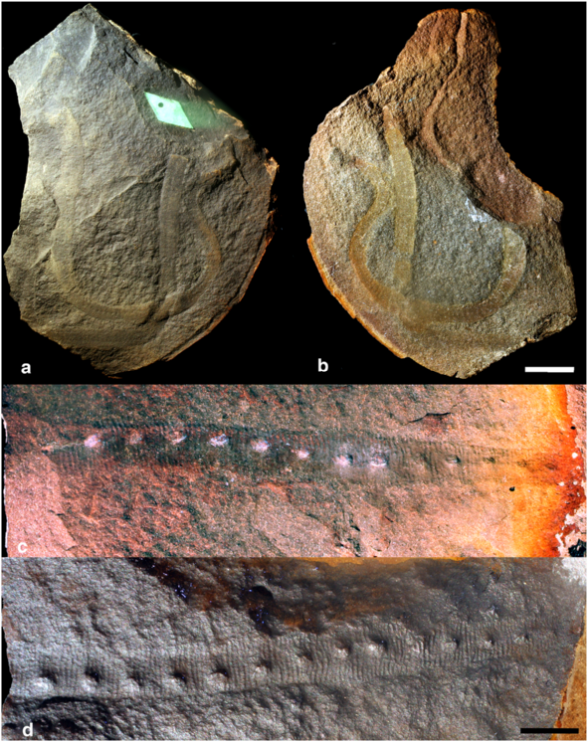
Type material of the genus *Ilyodes*, here proposed as a nomen dubium. **a**, **b** Type specimen of *Ilyodes elongata* Scudder 1890 (USNM PAL 38035), part (**a**) photographed using low angle incident light, from northwest; counterpart (**b**) photographed wet under polarised light. *I. elongata* is an elongate tube lacking any discernable limbs and is clearly not synonymous with *Helenodora inopinata*. Scale bar = 10 mm. **c**, **d** Type specimen of *Ilyodes divisa* Scudder 1890 (USNM PAL 38034), part (**d**) and counterpart (**c**), photographed wet under cross-polarised light. The parts of both specimens were previously coated in a dark ink, that we made no attempt to remove. *Ilyodes divisa* has a different body plan from *H. inopinata*, but has too few taxonomically useful characters to differentiate it from other fossil lobopodian taxa. Scale bar = 5 mm. High resolution versions of each photograph provided in additional materials (Additional file 1)

*Helenodora* has been designated as a junior synonym of *Ilyodes* Scudder 1890 [11], but we consider this to be incorrect. *Ilyodes* was first described as belonging to the Myriapoda [12], and some 90 years later *Helenodora inopinata* was described as an onychophoran-like animal [8]. The similarity of these taxa and as yet undescribed material from Montceau-les-Mines [13, 14], led to the suggestion that they were synonymous [15]. Scudder [12] erected two species of *Ilyodes*: *Ilyodes divisa* and *Ilyodes elongata.* Re-examination of the type material clearly demonstrates significant differences between *I. elongata*, *I. divisa* and *H. inopinata. Ilyodes elongata* is a long (min. 161 mm) and thin (max. 4 mm) parallel-sided tube bearing a large number of segments (up to 166) and lacking clear limbs (Fig. 1a, b; Additional file 1). This lack of similarity demonstrates that *I. elongata* is not synonymous with *H. inopinata*.

*Helenodora inopinata* and *I. divisa* are more directly comparable, sharing an annulated trunk with approximately the same number of annulae per limb-bearing segment (Fig. 1c, d; Additional file 1), but we argue that synonymising these taxa is incorrect for two reasons. First, that there are differences in the number of limbs: comparing similar length portions of the trunk, the holotype of *I. divisa* bears 15 limbs, and tapers in one direction, from a width of 5.5 to 3 mm, whereas, *H. inopinata* bears typically 20 limbs, is 6 mm wide at the widest point, and tapers in both directions. If we reconstruct *I. divisa* with a complete body of the same general proportions as *H. inopinata*, *I. divisa* would possess far more than the maximum of 20 walking limbs seen in any of the known material of *H. inopinata.* This evidence, that *I. divisa* bore a different number of limbs to *H. inopinata*, makes synonymy unlikely, but we cannot rule out the possibility. This brings us to the second, and more important reason not to synonymize: the holotype of *I. divisa* preserves scant evidence of its original anatomy – little more than trunk annulations and limbs – and thus possesses too few taxonomically informative characters for it to be reliably differentiated from other fossil lobopodian taxa. Consequently, we deem *Ilyodes divisa* to be a nomen dubium. Of the options available to rectify this situation, selection of a neotype from among Scudder’s material is not possible as he included only one specimen in this species. In theory, a neotype could be selected from subsequently published Mazon Creek lobopodians, but the simplest course of action, most likely to promote nomenclatural stability, is to limit the use of the name *Ilyodes divisa* to the holotype. *Helenodora inopinata* is thus the oldest available name for the taxon described in this paper.

We note also that the holotype of *I. elongata* is too incomplete to serve adequately as a name bearing type and *I. elongata* is thus also a nomen dubium. With no adequately diagnosed species, the genus *Ilyodes* should be considered as a nomen dubium.

#### Diagnosis

Same as for only species.

#### Type species

*Helenodora inopinata* Thompson and Jones from Francis Creek Shale, Carbondale Formation, Middle Pennsylvanian.

**Species*Helenodora inopinata*** Thompson and Jones 1980

#### Type specimens

Holotype: FMNH PE 29049. Paratype: FMNH PE 29050. Housed in the invertebrate fossil collections of the Field Museum of Natural History, Chicago, Illinois.

#### Material

**Fig. 2:**
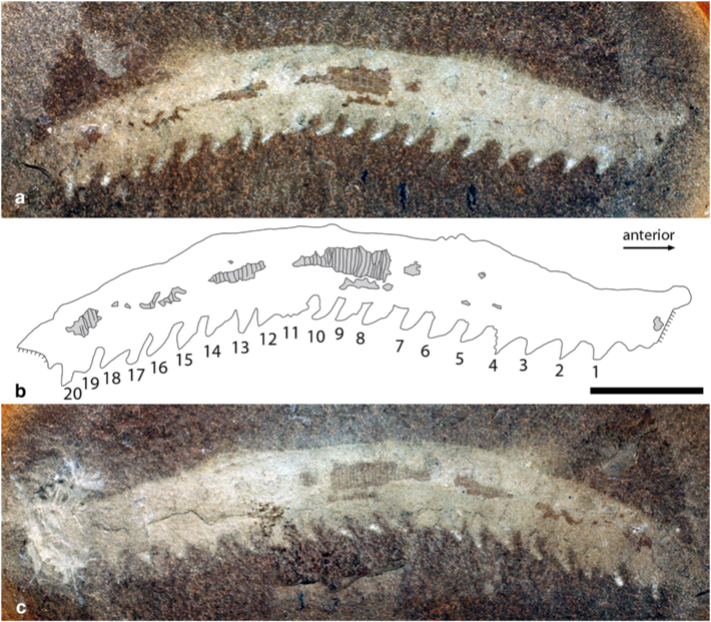
Holotype of *Helenodora inopinata* (FMNH PE29049). **a** Part and (**c**) counterpart, from the Francis Creek Shale, Westphalian D, Middle Pennsylvanian. Specimen preserved in lateral aspect and photographed wet under cross-polarised light. **b** Camera lucida drawing of part shows body outline and outline of limbs, as well as patches of annulated cuticle, shaded in grey. Note the dark patches outside the body margin ventral to limbs 5, 7 and 9. Details of both the anterior and posterior ends are not preserved, despite attempts to excavate the anterior of the counterpart (**b**) by Thompson & Jones [8]. Numbers refer to walking limbs from anterior to posterior. ann = trunk annulae. Scale bar = 10 mm. High resolution versions of each photograph provided in additional materials (Additional file 2)

**Fig. 3:**
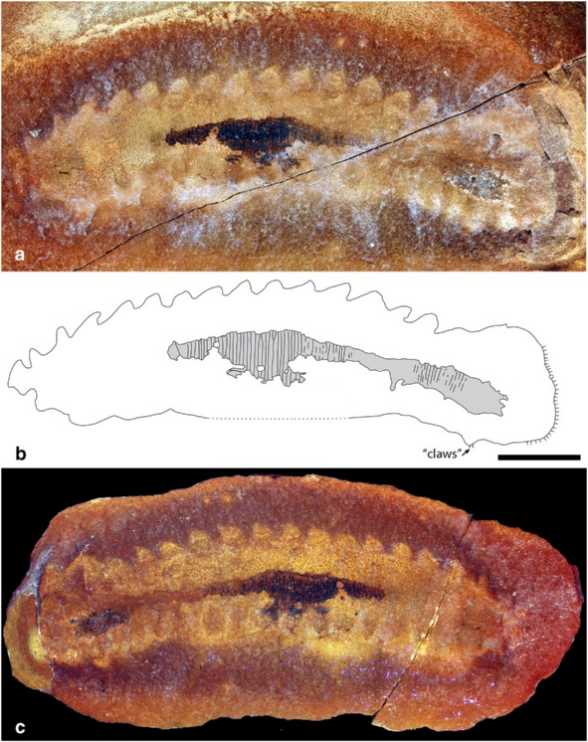
Paratype of *Helenodora inopinata* (FMNH PE29050). **a** Part and (**c**) counterpart from the Francis Creek Shale, Westphalian D, Middle Pennsylvanian. Specimen preserved in dorso-ventral aspect and photographed wet under cross-polarised light. **b** Camera lucida drawing of part shows body outline and outline of limbs, as well as patches of annulated cuticle, shaded in grey. Location of disputed claws indicated, further illustrated in fig. 6. Scale bar = 10 mm. High resolution versions of each photograph provided in additional materials (Additional file 3)

**Fig. 4:**
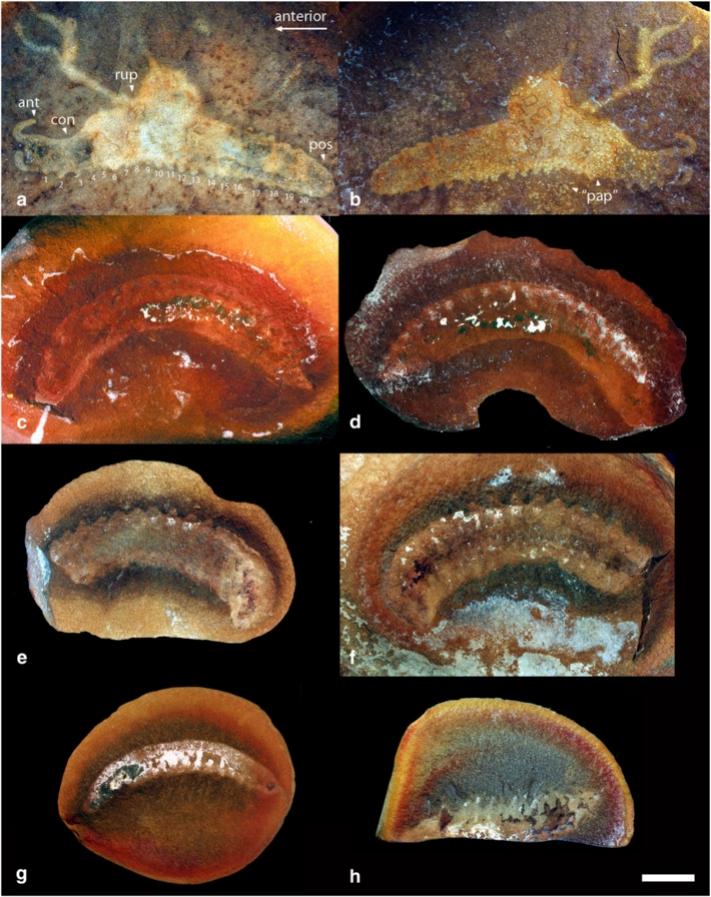
New specimens of *Helenodora inopinata* from the Francis Creek Shale, Westphalian D, Middle Pennsylvanian. **a**, **b** FMNH PE13966, part and counterpart. **c**, **d** FMNH 33380, part and counterpart. **e**, **f** FMNH PE33822, part and counterpart. **g** FMNH PE45049. **h** FMNH PE49784. Photographed wet under cross-polarised light. **a**, Specimen preserves complete anterior, defined by a constriction of the body outline (con), bearing frontal appendages (ant) as well as limbless posterior extension (pos) of the trunk. Ruptured body wall (rup) with putative escaped decaying tissues. Numbers refer to walking limbs from anterior to posterior (**b**). Putative dermal papillae (“pap”) visible both inside and outside of body wall. In all images, white or pale regions of the fossil correspond to a clay-filled (kaolinite) cast defining the overall morphology of the animal. Bright white patches on the surface of the nodules are caused by concentrations of kaolinite, not reflections or other artefacts associated with the photographic methods. The darker patches, which preserve details of external anatomy, are moulds of external anatomy preserved as siderite with patchy framboidal pyrite. The similarity of patches to features such as jaws (e.g. large dark patch at one end of (**g**)), is coincidental. Scale bar = 10 mm. High resolution versions of each photograph provided in additional materials (Additional file 4)

In addition to the type material (Figs. 2, 3; Additional files 2, 3), specimens considered to represent *H. inopinata* include: six new specimens described here, FMNH PE 13966, 33380, 33822, 45049, 49784 (Fig. 4; Additional file 4) and ROM 47513; two specimens figured by Haug et al. [10], ROM 45565 and 47978 (the latter also figured by Hay and Kruty [15] with the number NEIU MCP 184). As noted above, although unlikely we cannot completely exclude the possibility that the holotype of *Ilyodes divisa* (USNM PAL 38034; Fig. 1c, d) represents partial remains of *H. inopinata*, but it is too incomplete for this to be determined with any degree of certainty or for the taxon to be properly diagnosed. In addition, undescribed material from Montceau-les-Mines [13, Fig. 4d of 14] may be referable to this taxon.

#### Emended diagnosis

Elongated, vermiform fossils typically with 20 pairs of short, tapered legs arranged ventro-laterally and evenly spaced along the body, and one pair of unjointed frontal appendages; cuticle with fine annulations, approximately nine per segment.

### Revised description

**Fig. 5:**
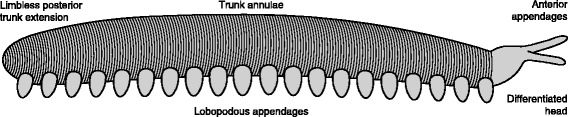
Reconstruction of *Helenodora inopinata*. Modified from Thompson & Jones [8] with the addition of differentiated head, frontal appendages, limbless posterior extension, and modification to limb morphology to reflect the new interpretations presented herein. Details of the cuticle of the limbs has been excluded as it is not preserved in the available material

The complete (or near complete) trunk ranges in length from 45 to 66 mm, and in width from 6 to 13 mm (with length:width ratios ranging from 4.9 to 9.3). The anterior portion of the trunk (head) bears a pair of unjointed frontal appendages (Fig. 5); where complete these are 4-5 mm long and 1.2 mm wide at their base (FMNH PE13966; Figs. 4a, b). Posterior of appendage pair 1 the trunk is constricted relative to the rest of the trunk (Figs. 2a, 4a, b, 5). Trunk annulae of the new material are consistent with those of previously described specimens, typically 9 per segment (where the complete preservation of limbs permits segment boundaries to be established). Walking legs have a mean length of 1.7 mm (n = 52), and mean width at the base of 2.1 mm (n = 50). A maximum of 20 pairs of walking legs is observed across all specimens, although the posterior-most appendages of the FMNH PE 29049 (Fig. 2) are incomplete and not clearly differentiated from the body outline). Specimens with a differentiated anterior and posterior of the trunk have 20 limb pairs definitively (FMNH PE 13966; 4a, b), giving a total of 21 appendage pairs (Fig. 5). Nevertheless, given the small number of complete specimens, we cannot exclude the possibility that some individuals bore a greater or smaller number of limbs. No cuticular features of the limbs are preserved, so the presence of limb annulation or ornament remains unknown. A short rounded extension of the trunk, posterior of the last appendage pair, is seen in the most complete specimens (Figs. 4a, b, 5).

### New anatomical interpretations

Prior to making interpretations of anatomy, it is first necessary to establish the framework within which interpretations are to be made. The fossils consist of a vermiform, annulated body with pairs of lobe-shaped, distally tapering projections. These lobe-shaped projections are generally paired (seven specimens; Figs. 3 and 4a, b, e, f, h) although only preserved as a single row in three specimens (Figs. 2 and 4c, d, g). We interpret this as evidence that the projections are paired and present on one surface of the body, one row being obscured in certain collapse orientations. The simplest interpretation of these structures is as paired lobose limbs. Their disposition in the fossils indicates that they are located closer to one surface of the trunk, and we take this to be the ventral surface, thus establishing the dorso-ventral axis. Thompson and Jones [8] postulated that the animal exhibited a greater degree of tapering in the anterior direction. The presence of paired frontal appendages allows us to corroborate their interpretation of the anterior-posterior axis.

A vermiform trunk with serially repeated, paired, non-segmented limbs, suggests *Helenodora inopinata* is best interpreted within the framework of the Palaeozoic lobopodians.

#### Head

Neither specimen described by Thompson and Jones [8] possesses a well-preserved head, contributing significantly to the uncertainty regarding the affinity of *H. inopinata*. The new material provides some additional information. We find good support for appendage pair 1 being frontal appendages. However, there is no consistent evidence that appendage pair 2 is differentiated from the remaining appendages, or for any structures resembling slime papillae. ROM 47978, figured by Haug et al. (2012), has a rounded swelling on one side of the head region posterior of the frontal appendages, and these authors interpreted this swelling as evidence of slime papillae. However, where the complete outline of the head is preserved (Fig. 4a, d) this structure is not, and in the absence of additional evidence, a taphonomic interpretation of the swelling as a post-mortem bulge in the cuticle is more parsimonious (see Taphonomy).

In the original interpretations, the possession of jaws was inferred because two specimens (FMNH PE 29049 and 29050; Figs. 2 & 3) were described as having a “…small dark patch in the approximate position of the jaw in living onychophorans.” [8]. In FMNH PE 29049 (Fig. 2) this small dark patch exhibits no relief and, based on EDX analysis, is chemically indistinguishable from other dark patches of the surrounding fossil, i.e. siderite with patchy framboidal pyrite. There is no evidence of preservation as carbon film, which we would expect in the remains of decay resistant structures such as jaws. We therefore interpret this dark patch in the same way as the other darker patches (see Taphonomy); it has no anatomical significance. In FMNH PE 29050 (Fig. 3) the ‘jaw’ is preserved as a small patch of pyrite with an indistinct shape. This patch is indistinguishable in composition from larger patches elsewhere on the fossil and from similar sized patches on the surface of the nodule outside the margin of the fossil (e.g. those visible in Fig. 2a ventral to limbs 5, 7 and 9). Furthermore, whatever the homologies of the limbs, it lies between the second and third preserved appendage pair, a location that is posterior to that of jaws in modern onychophorans, which develop between the cephalic lobe (which bears frontal appendages) and the segment which bears the slime papillae [16]. No comparable structures that might be interpreted as jaws are observed in other specimens, most notably FMNH PE 13966 (Figs. 4a, b) which has a complete anterior. Given all this, we can find no convincing evidence for the presence of jaws in *H. inopinata*.

#### Trunk

Trunk (dermal) papillae have been described on several specimens, by Thompson and Jones [8] and Haug et al. [10]. However, these putative dermal papillae are not systematically arranged in rows as would be expected, and the material presented here (most notably FMNH PE 13966; Figs. 4a, b) illustrates that the papilla-like structures are found outside of the annulated regions and even outside of the body outline, although here they are less dense. We cannot totally reject the possibility that some of the features are dermal papillae, but the evidence for them is equivocal at best, and they should not be considered a diagnostic feature. ROM 47978 (figured by Hay and Kruty [15] as NEIU MCP 184) is described as possessing tubercular bosses on the frontal margins of the segments, which are clearly visible as dark circular patches <1 mm across [10].

#### Legs

**Fig. 6:**
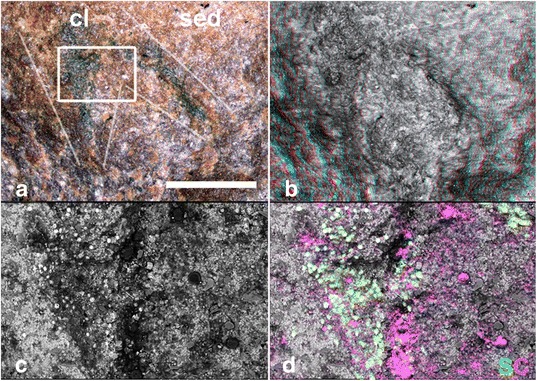
Putative claws of *Helenodora inopinata* (FMNH PE29050, counterpart). **a** Optical image produced using Alicona Infinite Focus microscope, with boundaries of putative claws demarked. **b** anaglyph stereo image to show 3D form of surface. **c** Back-scattered electron micrograph of region marked on optical image. **d** Selected element maps generated using EDX analysis and false coloured for comparative purposes. ‘Claws’ are represented by framboidal structures rich in sulphur and iron (pyrite), with some organic carbon. The superficially claw-like morphology is exaggerated by the shape of the broken surface of the fossil, clearly visible in the anaglyph stereo image. sed = sediment. cl = putative claws. Scale bar = 100 μm

Thompson and Jones [8] described claws at the tips of seven limbs in FMNH PE 29050 (Fig. 3), one pair of which they figured. The remainder are less clear, and all are restricted to a single leg row. There are no claws visible on any other specimens. The claws of FMNH PE 29050 (Fig. 3) appear as small dark patches with an overall elongate shape, and EDX analysis reveals that the pair illustrated [8] is composed of a thin film of carbon and framboidal pyrite (Fig. 6). Patches of comparable size, shape and composition are seen across this and other specimens, in locations that are inconsistent with an interpretation as claws (some located outside the boundaries of the fossil). Thus it seems likely that the ‘claws’ in FMNH PE (Fig. 3) are nothing more than a fortuitously located patch of pyrite.

### Taphonomy

The Mazon Creek Lagerstätte has yielded a wide array of animal and plant fossils with soft part preservation [17]. These fossils almost exclusively occur within siderite nodules as external moulds, generally showing signs of collapse but in many cases retaining a degree of their original three-dimensionality. Minerals are often present on the internal surfaces of the moulds, and may fill the entire cavity (i.e. forming a cast). These minerals include pyrite, calcite, sphalerite and, most commonly, kaolinite [18]. The exceptional preservation of this biota is thought to reflect rapid burial and early formation of concretions, preventing scavenging and aerobic decomposition and protecting fossils from subsequent compaction [19]. Here we consider the taphonomic history of specimens of *Helenodora inopinata*, and the implications of this for anatomical and phylogenetic reconstruction, based on two lines of evidence: patterns of character loss in decay experiments, and the preservation of characters in Mazon Creek fossils that we would expect to have comparable decay resistance to characters in onychophorans.

#### Preservation

**Fig. 7:**
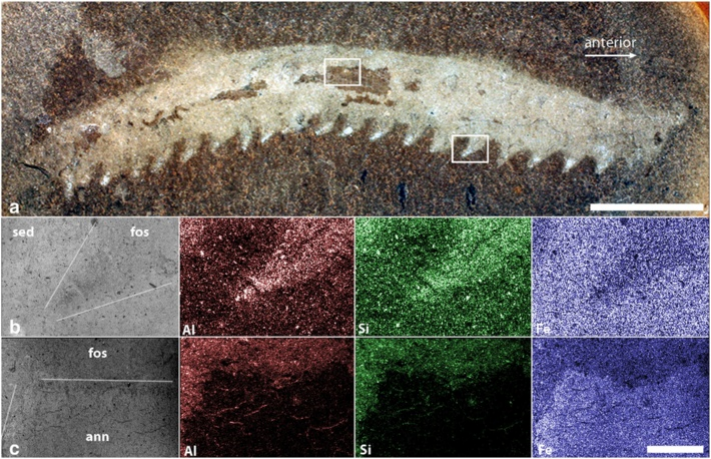
Preservation of *Helenodora inopinata* (FMNH PE29049, part). **a** Specimen photographed wet under cross-polarised light, with locations of element maps highlighted. Scale bar = 10 mm (**b**, **c**) Back-scattered electron micrographs with selected element maps generated using EDX analysis and false coloured for comparative purposes. **b** Limb, triangular structure in the top and left of the image, is richer in aluminium and silica but poorer in iron than the surrounding nodule, suggesting replacement by clay minerals, concentrated at the tip of the limb. sed = sediment. fos = fossil. **c** Boundary between annulated central region and remainder of fossil. Annulated region is on the bottom left of the field of view, and is richer in iron and poorer in aluminium and silica compared to the remainder of the fossil; the bulk composition is indistinguishable from the siderite nodule with the addition of patchy framboidal pyrite. ann = annulated region. fos = remainder of fossil. Scale bar = 1 mm

Specimens of *H. inopinata* are preserved in a manner consistent with other soft-bodied Mazon Creek fauna [18]. The fossils are largely flattened, but retain some three-dimensionality and have some relief relative to the broken surface. Where the fossils are preserved as convex surfaces, features such as limbs are preserved in positive relief and the dark annulated patches (Fig. 2) are proud of the surface of the split, with surface anatomical features (annulae and putative dermal papillae) preserved in positive relief. Correspondingly, concave fossils have features preserved in negative relief with the dark annulated patches below the level of the split. In specimens with both part and counterpart preserved, features positive in the part correspond to negative features in the counterpart (and vice versa). We interpret this as evidence that the fossils were preserved as an external mould of the surface of the outer cuticle, later filled with clay minerals to form a cast. Generally the nodules split through the cast, giving a fossil with the body outline with no external characteristics other than shape, and no internal anatomy. In a few specimens the plane of the split is coincident with the outer surface of the cast and exposes a mould of the outer surface of the animal, preserved as dark patches of siderite and pyrite. The body outline of each specimen (most clearly visible when wet and photographed using cross-polarised light) is marked by a pale colouration, and a smoother texture than the matrix. EDX analysis suggests that pale areas of the fossil are relatively richer in silica and aluminium (but not magnesium, calcium or potassium), and poorer in iron than the surrounding nodule (Fig. 7). This is consistent with preservation as kaolinite, which is often associated with Mazon Creek fossils [20, 21]. This is most clearly expressed as patches of material toward the distal portion of some of the limbs which appear bright white under incident illumination (e.g. PE29049, Figs. 2 & 7). Despite their position on the limbs, these patches are not consistent with an interpretation as claws: they vary considerably in size and shape, covering the majority of the limb in some cases, and are always *within* the margin of the limb. EDX analysis of these white patches shows a stronger, but otherwise comparable, signal to the remainder of the fossil (i.e. richer in aluminium and silica, poorer in iron), and they are indistinguishable from similar white regions along the midline of the trunk (e.g. between darker annulated patches, described below). The cause of this concentration of clays in some limbs is not entirely resolved, but we interpret it as a taphonomic artefact, either reflecting greater retention of three-dimensionality in the distal regions of the limbs leaving a more extensive void which was infilled by clay, or a simple consequence of the orientation of the split through the nodule.

A number of specimens exhibit patches of darker material on the outer surface of the cast, preserving superficial features of the cuticle (annulae and putative dermal papillae). These patches have the same composition as the siderite nodule (Fig. 7) and some specimens (e.g. the paratype, FMNH 29050; Fig. 3) bear extensive, but patchy, framboidal pyrite. Small patches (<1 mm across) of framboidal pyrite, associated with organic carbon, occur across the broken surface of the nodule, but are not restricted to the fossil.

Taking this evidence of preservation of *H. inopinata* together suggests rapid burial of the animal followed by collapse prior to nodule formation, resulting in a largely flattened, but 3d fossil (i.e. we interpret the flattening of the fossils to be primarily the result of decay and collapse of the body, not compaction of the nodule). Decay of the body tissue left an external mould and a void within which kaolinite precipitated, either directly or through alteration of illite by migrating pore fluids [22]. It is not clear from the evidence in these fossils whether the pyrite formed early, replacing or coating parts of the original cuticle, or late as void-fill in a similar manner to the clays.

#### The role of experimental decay

Thompson and Jones [8] consider the preservation quality of *H. inopinata* to be good, with little decay having occurred prior to preservation, stating: “The specimens of *H. inopinata* are not poorly preserved as the body outlines are clear and the cuticle is preserved in places complete with annulations and papillae.” (p 593). However, these features are preserved as moulds of the external surface of the carcass, rather than as organic carbon films derived from the cuticle.

The results of decay experiments with onychophorans [23] allow the degree of decay of *H. inopinata* specimens, prior to preservation, to be assessed more rigorously. Several features of *H. inopinata* are comparable to onychophorans in an advanced state of decay [23]: trunk annulae are only patchily preserved, and limb cuticle lacks both annulae and ornament; the black mineral ‘film’ marking the outer margin of the fossils is incomplete. In one specimen (FMNH PE13966; Figs. 4a, b) the body outline is extended as an irregular stain, which compares to advanced decay of onychophorans, in which rupture of the outer cuticle results in extrusion of body cavity contents.

Given this evidence for significant decomposition of *H. inopinata*, can we differentiate between characters that were lost to decay and characters which were never present? This is potentially informative regarding phylogenetic placement, and jaws and claws are of particular significance in this context: they played an important part in the original interpretation of *H. inopinata* as an onychophoran [8], and also constitute potential synapomorphies of the total group Onychophora (e.g. Smith and Ortega-Hernandez [2]). In addressing the question of character loss it is important that we take into account not only patterns of decay, but also how preservation has acted to preserve characters. In order to do this we consider the taphonomy of onychophoran jaws and claws, and other features, in a comparative context: we assess the likelihood of preservation on the basis of whether features in other fossils that have similar resistance to decay are preserved in the Mazon Creek Lagerstätte.

As *H. inopinata* is a member of Panarthropoda, if it originally possessed jaws and claws they would have been composed of sclerotised chitin, and if modern onychophorans are a good analogue for *H. inopinata*, jaws and claws would be expected to be among the most decay resistant characters [23]. The preservation potential of jaws and claws should be comparable to the chaetae of polychaete annelids and to the cuticle of euarthropods, taxa that are common in the Mazon Creek: like jaws and claws, both chaetae and cuticle are composed of sclerotised chitin, and both are known from experimental analysis to be decay resistant [24, 25]. Thus we would expect that if polychaete chaetae and arthropod cuticle are commonly preserved in the Mazon Creek biota, and preserved in a consistent way, so would be the jaws and claws of *H. inopinata*. This is not what we find. Polychaete chaetae and arthropod cuticle are common, preserved usually as distinct molds and rarely with any original material present [18, 26]. The putative jaws and claws in *H. inopinata*, however, are preserved in only a few specimens, and these equivocal traces are not preserved as distinct molds but as indistinct dark patches associated with organic carbon films and framboidal pyrite, generally lacking relief. The most parsimonious interpretation of these taphonomic data, and the lack of any compelling anatomical evidence, is that the absence of jaws and claws in *H. inopinata* reflects original morphology, not taphonomic loss (i.e. *H. inopinata* did not have claws or jaws).

Specimens of *H. inopinata* preserve aspects of the external morphology of the cuticle as external molds sometimes with a pyrite film. The fidelity of preservation of trunk annulae, and putative dermal papillae, coupled with the presence of frontal appendages, or at least their outer cuticle, is in contrast with the lack of slime papillae (a key apomorphy of the onychophoran crown and indicator of a terrestrial life habit). Decay experiments suggest that slime papillae should follow a similar taphonomic pathway to frontal appendages and other structures essentially dictated by the integrity of the cuticle. The absence of slime papillae in *H. inopinata* can thus be interpreted in one of two ways. Either they were originally present but unlike extant onychophorans had a different decay pathway to that of other cuticular structures that are preserved (presumably reflecting fundamentally different composition and histology); or they were genuinely absent. The latter interpretation is the more parsimonious.

Experimental taphonomy [23] suggests that distortion of the body outline of onychophorans commences early in the process of decay. Given the congruence of other features of *H. inopinata* with the decay data (described above) we suggest that the fossil specimens represent the remains of an organism in an advanced state of decay, and that the shape of the limbs and trunk is unlikely to preserve the original morphology. Rather, the outer cuticle will have swollen, whilst the epidermis would have shrunk relative to the condition *in vivo*. This is supported by the variable length:width ratio of the fossils, and by the comparatively slender trunk of the specimen exhibiting rupture of the cuticle, the outline of this specimen arguably better reflecting the body shape before decay.

### Affinity

The inclusion of all the material presented here into a single taxon (*Helenodora inopinata*) is supported by a number of shared characters: the overall size and shape of the trunk is consistent across the specimens, as is the general limb morphology; no more than 20 walking limb pairs, with an additional pair of anterior frontal appendages, are consistently seen, and all complete specimens are in accord with this interpretation; where preserved, the cuticle bears fine annulations (8 or 9 per segment); no significant posterior extension (or ‘tail’) is visible in any of the specimens, beyond a short rounded termination (Fig. 5). Not all specimens exhibit all features, due to the rare preservation of the anterior and posterior of the animal, and differing taphonomy of the specimens. However, the character distributions of these specimens overlap to a degree that suggests they are a single taxon. An unnamed taxon from Montceau-les-Mines, described as “virtually identical” to *H. inopinata* [13] will likely also be included within this genus, but establishment of synonymy awaits a full description*.*

Thompson and Jones [8] concluded that an onychophoran affinity was most consistent with their evidence, identifying: lobose limbs, paired terminal claws, an annulated trunk with dermal papillae and general similarity of size and shape to modern onychophorans. This led the original authors to tentatively conclude that “*H. inopinata* can be classified with the living onychophora”, and to postulate a reconstruction with ambiguous head and tail morphology. A recent phylogenetic analysis of a range of panarthropod taxa [4] supports this hypothesis, recovering *H. inopinata* (their *Ilyodes*) in an unresolved clade consisting of three extant onychophoran species and the Eocene taxon *Tertiapatus* supported by two synapomorphies: slime papillae (char. 15, state 1) and pre-oral appendages (char. 24, state 1), see also Additional file 5 and (character key in Additional file 6). In light of the new material and our redescription this discussion should be revisited. Here we modify the character coding for *H. inopinata* of Yang et al. [4] to reflect both our new anatomical and taphonomic interpretations (for details, Table 1 and Additional files 7 and 8). We describe previously unknown anatomy of trunk annulations (chars. 36, 38), limbs (char. 67) and a limbless posterior extension of the trunk (char. 75). The absence of jaws (chars. 12-14), claws (chars. 46, 64-66, 80) and slime papillae (char. 15) are here interpreted as real, based on the arguments articulated above. In addition, on the basis of similar reasoning regarding preservation potential, we recode decay prone characters that are not preserved in *H. inopinata* as ambiguous: mouth position (char. 24), midgut glands (char. 52) and dermal papillae (char. 62).

**Table 1:** Summary of changes to data matrix for phylogenetic analysis

New anatomical interpretations		
#36	(?) - > (1)	Annulations
#38	(?) - > (0)	Annulations
#67	(?) - > (0)	Limbs
#75	(?) - > (1)	Posterior
Ambiguity due to lack of preservation		
#24	(1) - > (?)	Mouth position
#52	(0) - > (?)	Midgut glands
#62	(1) - > (?)	Dermal papillae
Genuine absence of decay resistant characters		
#12	(?) - > (0) ; nc	Jaws
#13	(?) - > (-) ; nc	Jaws
#14	(?) - > (0) ; nc	Jaws
#15	(1) - > (0) ; (1) - > (?)	Slime papillae
#46	(?) - > (-) ; nc	Claws
#64	(1) - > (0) ; (1) - > (?)	Claws
#65	(0) - > (–) ; (0) - > (?)	Claws
#66	(1) - > (–) ; (1) - > (?)	Claws
#80	(?) - > (-) ; nc	Claws

Three different types of modifications to the character coding for *Helenodora inopinata* (=*Ilyodes*), based on data presented by Yang et al. [4] with their character numbers used. Modifications refer to both coding methodologies unless two changes are shown separated by a semi-colon, in which case the first refers to the decay-informed coding, the second to conservative coding, nc = no change to matrix. The aspect of the anatomy of *H. inopinata* in question is reported for reference. Complete data matrix presented in Additional file 8 (character key in Additional file 6)

**Fig. 8:**
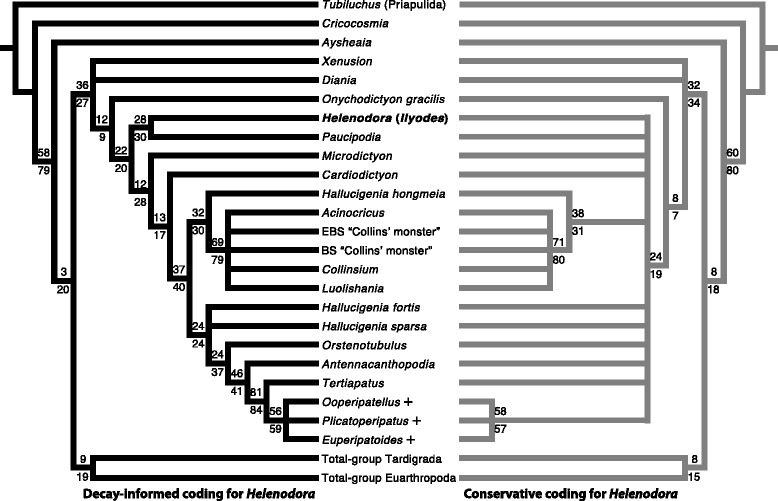
Amended panarthropod phylogeny. Strict consensus of 57 (left) and 147 (right) most parsimonious trees under implied weights (k = 4), + denotes extant onychophoran taxa. Arthropod and tardigrade total-groups collapsed for clarity, as they are unchanged from the results presented by Yang et al. [4]. Left-hand topology derived from modifying the character coding for *Helenodora inopinata* (=*Ilyodes*) of Yang et al. [4] to reflect the new observations herein. Right-hand topology derived in the same way but with characters whose absence is inferred from taphonomic analysis coded as ambiguous. For each dataset, the consensus topology for total-group Onychophora was stable with all but the most stringent concavity values (equal weights and implied weights with concavity constants in the range of k = 4 to ∞). Under stricter homoplasy penalization (k < 4) the position of *Aysheaia* and *Onychodictyon ferox* are unstable, reducing the resolution, but the position of *H. inopinata* relative to crown-Onychophora is unchanged. Numbers on nodes indicate node-support based on symmetric resampling, under equal (above) and implied weights (below, k = 4). The results shown are difference in frequencies, given as ‘GC’ values (for Group present/Contradicted [35]). For clades recovered in both analyses, their support is comparable. When taphonomic evidence is used to inform character coding *H. inopinata* is resolved as a stem-onychophoran, i.e. clearly outside of crown-onychophora, contra Yang et al. [4]. If taphonomic evidence is not used the position of *H. inopinata* is ambiguous and much of the resolution within total-group Onychophora is lost, demonstrating the utility of experimental and comparative taphonomy in phylogenetic reconstruction.

Using our amended data, *H. inopinata* is resolved a stem-onychophoran, sister to *Paucipodia*, unequivocally outside crown-Onychophora (Fig. 8). *H. inopinata* and *Paucipodia* are united by two synapomorphies: homonomous trunk annulations (char. 38, state 0) and a lack of metamerically arranged dorsolateral epidermal specializations (char. 39, state 0), see also Additional file 5 (character key in Additional file 6). A lack of terminal claws (char 64, state 0) is autapomorphic for *H. inopinata*. We recover the three extant onychophorans as a clade (crown-Onychophora) with a single synapomorphy: differentiated distal foot in lobopodous trunk limbs (char. 67, state 1), with the Eocene *Tertiapatus* as their immediate sister taxon.

To examine the affect of using taphonomic evidence and the results of decay experiments to inform interpretations of anatomy, we repeated the analysis except for coding as ambiguous those characters whose absence is interpreted on the basis of our taphonomic analysis (jaws, claws and slime papillae). This produces an almost entirely unresolved onychophoran total-group (Fig. 8), with *H. inopinata* acting as a rogue taxon occupying several different but equally parsimonious phylogenetic positions. If *H. inopinata* is pruned from the consensus the left-hand topology of Fig. 8 is recovered. It is not simply the case that added uncertainty leads to the collapse of the least-well supported clades (Fig. 8). Rather, the loss of resolution can be directly attributed to the ambiguity added by coding jaws, claws and slime papillae as uncertain rather than absent in *H. inopinata*.

It is worth noting in this context that the position of *H. inopinata* at the base of the onychophoran stem is not the result of stemward slippage [27] caused by loss to decay of the synapomorphies shared by more derived onychophorans. In decay experiments, the sequence of character loss in onychophorans does not correlate with phylogenetic informativeness [16], and several of the synapomorphies are decay resistant – had they been present in *H. inopinata* we would expect evidence of them in the fossils. This analysis demonstrates the utility in phylogenetic reconstruction of comparative taphonomic analysis informed by evidence of experimental decay: it has the potential to resolve equivocal character codings and thus reduce the negative impact of incomplete fossil data on phylogenetic resolution and placement of otherwise controversial fossil taxa.

### Macroevolution

The Mazon Creek Lagerstätte is intermediate in age between the Cambro-Ordovician lobopodian-bearing faunas (the unnamed specimen described by Whittle et al. [28] being the youngest) and Cretaceous and Tertiary onychophorans preserved in Dominincan and Baltic amber [6, 7]. During this interval, the lineage leading to modern Onychophora made the transition from marine to terrestrial habitats, but the timing of this transition is poorly constrained. Divergence time estimates between the modern groups of onychophorans (Peripatidae and Peripatopsidae), calibrated to be older than the break-up of Pangea (142 Ma, McLoughlin [29]), place the origin of crown-Onychophora in the Lower Devonian, but with 95 % credibility intervals (depending on the data used in the analysis) ranging from the Upper Ordovician to the Carboniferous [30]. Considering the terrestrial habit of all extant onychophorans, terrestrialisation is likely to have occurred prior to this event. The Mazon Creek represents a spectrum of depositional environments ranging from brackish estuarine to marginal marine with both terrestrial and marine components. The fauna exhibits a range of life habits [21], and despite a dominance of pelagic or nektonic marine taxa, the collecting site from which most *Helenodora inopinata* specimens come (Pit 11) also preserves benthic organisms (bivalves, shrimp, holothurians) as well as a terrestrial component [31]. (For several *H. inopinata* specimens precise locality data are unknown). Given that the biota is ecologically mixed, the organisms associated with *H. inopinata* from Pit 11 provide few constraints on its life habits and we are limited to anatomical evidence.

Anatomically, *H. inopinata* is similar to modern onychophorans in some respects, including overall size and shape, number of limbs, lack of sclerites and homonomous trunk annulation. But *H. inopinata* lacks any clear anatomical evidence of a terrestrial habit, such as unequivocal dermal papillae or slime papillae. Thus we conservatively interpret *H. inopinata* as being aquatic, and for the reasons outlined above we cannot exclude the possibility that, like their Lower Palaeozoic counterparts, Carboniferous members of the onychophoran total group were marine.

The presence of *H. inopinata*, along with the recently described *Carbotubulus* Haug et al. [10], in the Pennsylvanian Francis Creek Shale extends the range of aquatic lobopodian-grade animals into the Carboniferous, and is consistent with a post-Carboniferous origin of terrestrial onychophorans. However, in contrast to *Carbotubulus*, aspects of the morphology of *H. inopinata*, a member of the total-group Onychophora, and its occurrence in nearshore or estuarine deposits (rather than the fully marine settings of earlier Palaeozoic lobopodians) may reflect an intermediate ecological stage in onychophoran evolution, prior to the advent of terrestrialization in the later onychophoran stem.

## Conclusions

We propose that *Ilyodes* Scudder is a nomen dubium because the type and only specimens of the two species assigned by Scudder to the genus do not possess the most diagnostic parts of the body (i.e. head, posterior and, in the case of *I. elongata*, limbs) and are too poorly preserved to differentiate them from other fossil lobopodians. Much more of the anatomy of *Helenodora inopinata* is now known, including details of the anterior and posterior of the trunk, allowing a more informed interpretation of its affinity. Our phylogenetic analysis demonstrates the value of taphonomically informed interpretations of anatomy, taking decay patterns into account, in improving resolution of relationships. *Helenodora inopinata* is a stem-onychophoran, with close affinity to the Cambrian *Paucipodia*, lacking claws, jaws and slime papillae. This taxon, along with *Carbotubulus* demonstrates the continued existence of aquatic, lobopodian-grade onychophorans into the late Palaeozoic and, possibly, a post-Carboniferous origin of the terrestrial onychophoran crown.

## Methods

Alongside the material described by Thompson and Jones [8] as *Helenodora inopinata* (FMNH PE 29049 & 29050; Figs. 2 and 3 respectively), seven subsequently discovered specimens were examined, FMNH PE 13966, 33380, 33822, 45049, 49784 (Fig. 4) and ROM 45565 & 47513, alongside illustrations of ROM 47978 presented in Haug et al. [10]. Specimens are housed at the Field Museum, Chicago (FMNH) and the Royal Ontario Museum, Toronto (ROM), and examined while on loan in Leicester with permission from the respective museums. These 10 specimens of *H. inopinata* were compared with the holotypes (and only specimens) of *Ilyodes divisa* Scudder (USNM PAL 38034; Figs. 1c, d) and *Ilyodes elongata* Scudder (USNM PAL 38035; Figs. 1a, b), held in the US National Museum, Washington DC.

Specimens PE 29049, PE 29050, PE 33822, PE 45049, ROM 47978 and ROM 47513 come from Peabody Coal Company, Pit 11, Will County, Illinois, USA. Specimens PE 13966 and PE 49784 come from the same locality, with additional information known regarding their precise horizon: PE 13966, T31N R9E 6 NE 1/4, SE ¼ Kankakee County; PE 49784, Baird Locality # 263, POND P T31N, R9E 8 SW 1/4, SW 1/4, SW ¼, Kankakee County. PE 3380 comes from a strip mine of Grundy – Will Counties. ROM 45565 also comes from the Mazon Creek area, but no precise locality information is known.

Phylogenetic analysis was performed on a modified version of the data matrix presented by Yang et. al [4] following the same methodology as the original authors: analysis was run in TNT [32] under New Technology Search, using Driven Search with Sectorial Search, Ratchet, Drift, and Tree fusing options activated in standard settings [33, 34], data matrix and search settings given in Additional file 8. The analysis was set to find the minimum tree length 100 times. All characters were treated as unordered. Analysis was performed under equal weights and under implied weighting, with a range of concavity values (k = 1, 3, 4, 200) to assess the affect of differing homoplasy penalization. Of a number of recent phylogenetic analyses of panarthropods we chose the Yang et al. [4] matrix as the basis for our analysis because, in addition to a broad range of lobopodian taxa, it contains three different extant onychophorans, thus allowing us to test the hypothesis that *H. inopinata* lies within crown-Onychophora. Two different coding strategies were used, both of which incorporate our new observations: first, a ‘conservative’ approach in which character interpretations contingent on taphonomic evidence were coded as ambiguous; second, where decisions about character interpretations were based on analysis of the taphonomy of *H. inopinata* informed by the results of experimental decay of onychophorans [23]. This allowed us to test the impact of taphonomically informed character codings on phylogenetic results. A summary of our alternative codings in presented in Table 1, full details in Additional file 7. Clade support was estimated using symmetric resampling implemented in TNT [35], one hundred replicates were performed conducting a new technology tree search consisting of 2 Wagner trees (with random addition sequences) followed by TBR (saving 10 trees per replicate) and 10 cycles of ratchet.

Specimens were photographed using a Canon digital SLR camera with a polarising filter, and RGB levels of resulting images were adjusted using Adobe Photoshop to better show the fossils. Textural and compositional data regarding preservation of anatomical characters were collected using a Hitachi S-3600 N Environmental Scanning Electron Microscope with Oxford INCA 350 EDX system (Department of Geology, University of Leicester), providing elemental mapping and point-and-ID spectra. Partial pressure was 20 Pa, working distance was between 9-12 mm, with an operating voltage of 15kv. Specimens were uncoated.

## Additional files

Additional file 1:
**Full resolution versions of images presented in Fig.** **1a–d** **.** RGB levels have been adjusted to better show the fossils, in some cases only in the region of the nodule that bears the fossil. For further details see caption at Fig. 1. (PDF 86713 kb) Additional file 2:
**Full resolution versions of images presented in Fig.** **2a,c** **.** RGB levels have been adjusted to better show the fossils, in some cases only in the region of the nodule that bears the fossil. For further details see caption at Fig. 2. (PDF 51834 kb) Additional file 3:
**Full resolution versions of images presented in Fig.** **3a,c** **.** RGB levels have been adjusted to better show the fossils, in some cases only in the region of the nodule that bears the fossil. For further details see caption at Fig. 3. (PDF 53604 kb) Additional file 4:
**Full resolution versions of images presented in Fig.** **4a–h** **.** RGB levels have been adjusted to better show the fossils, in some cases only in the region of the nodule that bears the fossil. For further details see caption at Fig. 4. (PDF 219449 kb) Additional file 5:
**Amended panarthropod phylogeny with synapomorphies mapped onto consensus tree topology.** Upper topology derived from modifying the character coding for *Helenodora inopinata* (=*Ilyodes*) of Yang et al. [4] to reflect the new observations herein. Lower topology derived in the same way but with characters whose absence is inferred from taphonomic analysis coded as ambiguous. For details of how trees were generated see Methods and caption of fig. 8. Upper numbers refer to character number and lower (bracketed) numbers refer to character state, both from analysis of Yang et al. [4]. Grey boxes indicate synapomorphies not present in all trees used to draw the consensus. (PDF 369 kb) Additional file 6:
**Character coding.** Character descriptions and character states from Yang et al. [4], for those characters modified in this analysis (Table 1, Additional file 7), and/or those characters mapped as synapomophies (Additional file 5). (PDF 54 kb) Additional file 7:
**Full details of modifications to the character coding for** ***Helenodora*** **inopinata (=** ***Ilyodes*** **).** Based on data presented by Yang et al. [4]. Changes highlighted in bold. (PDF 45 kb) Additional file 8:
**Character matrix and** ***TNT*** **search parameters.** Based on data presented by Yang et al. [4], with the addition of our new coding for *Helenodora* inopinata and our ‘conservative’ coding ignoring taphonomic data. Changes described in the text. (TXT 6 kb)
