# Supplementary figures and images for: The impact of taphonomic data on phylogenetic resolution: Helenodora inopinata (Carboniferous, Mazon Creek Lagerstätte) and the onychophoran stem lineage
Source: BMC Evol Biol. 2016 Jan 22;16:19. doi: 10.1186/s12862-016-0582-7 (PMC4722706; doi:10.1186/s12862-016-0582-7)

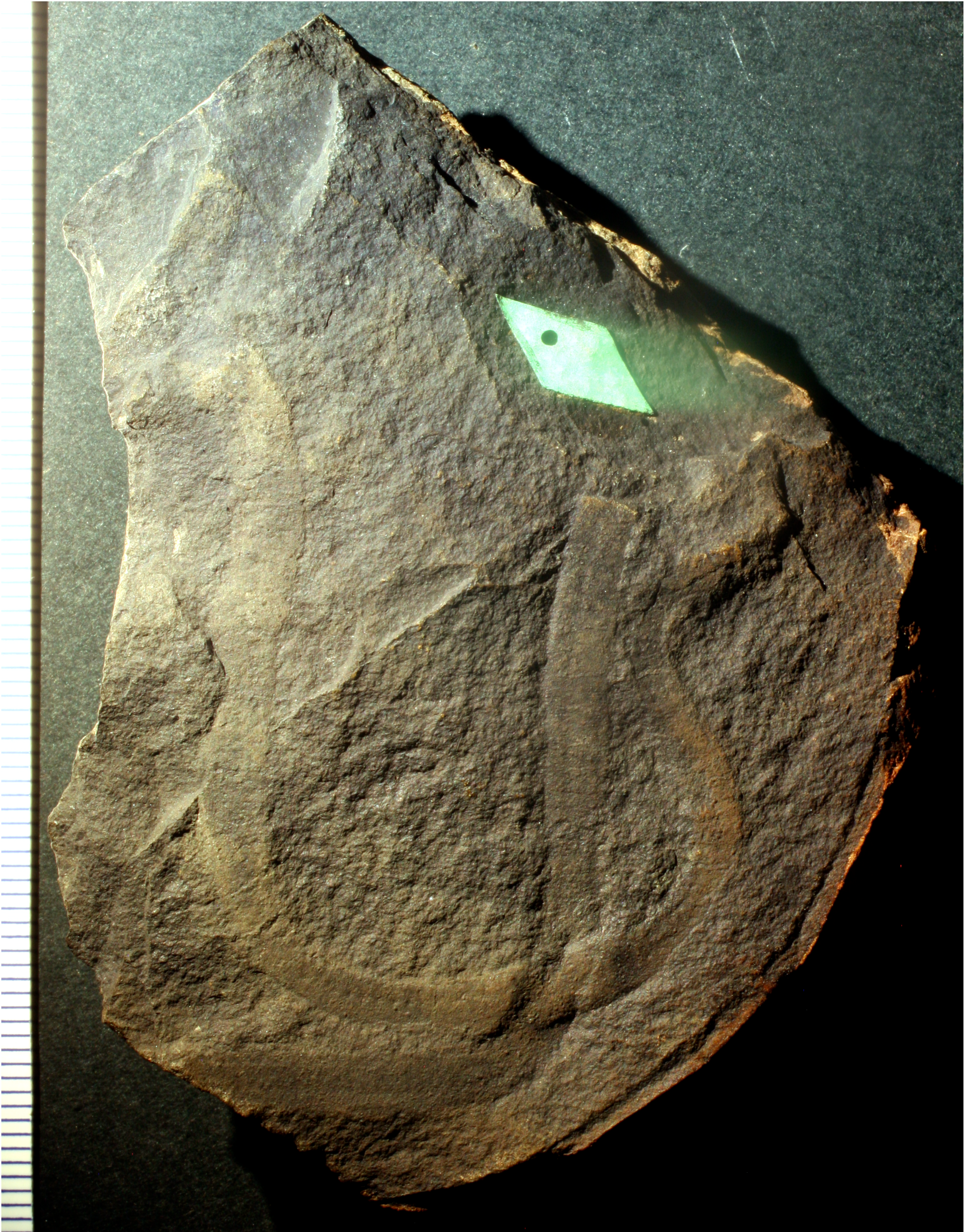

FIG 1A FULL

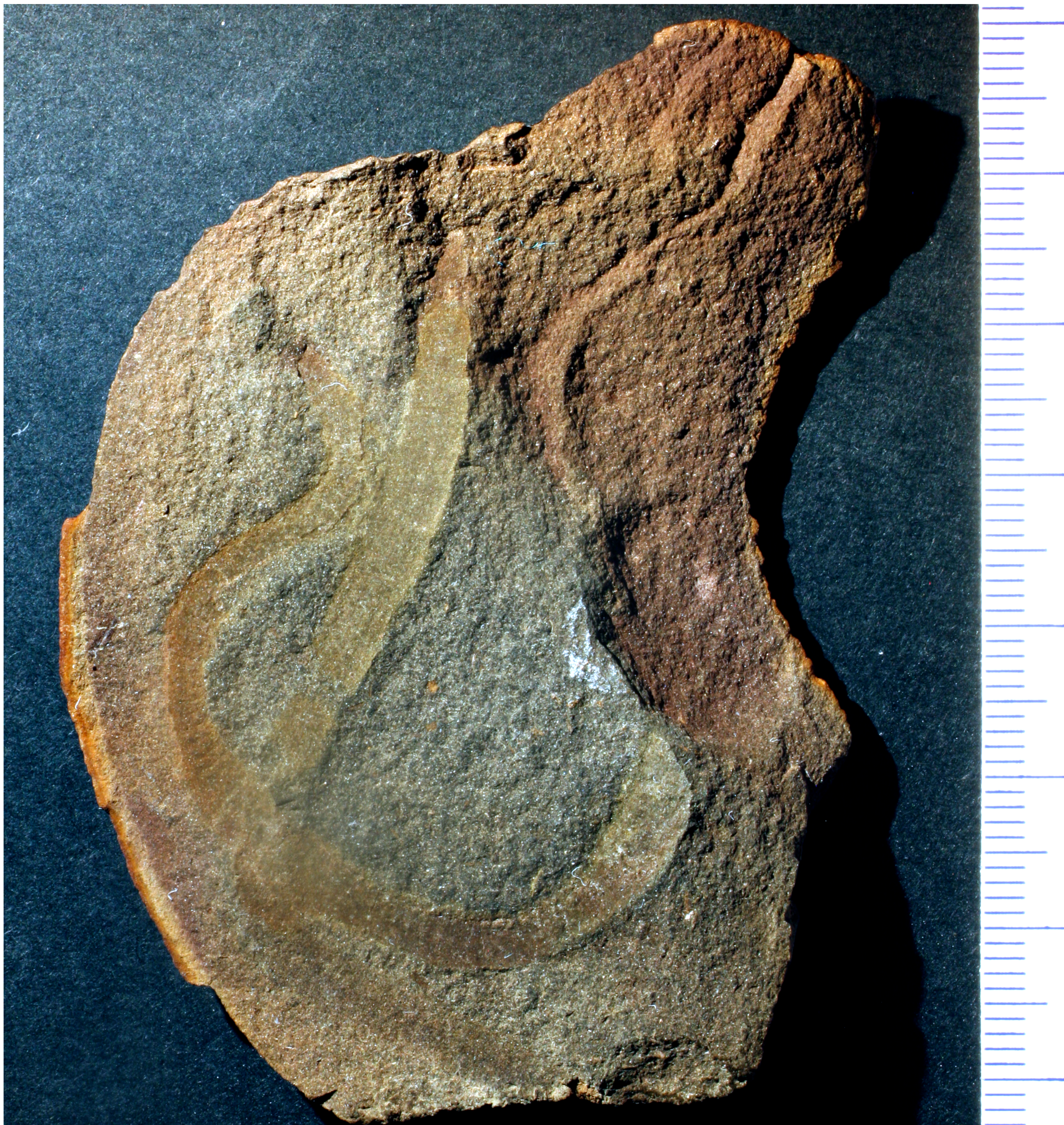

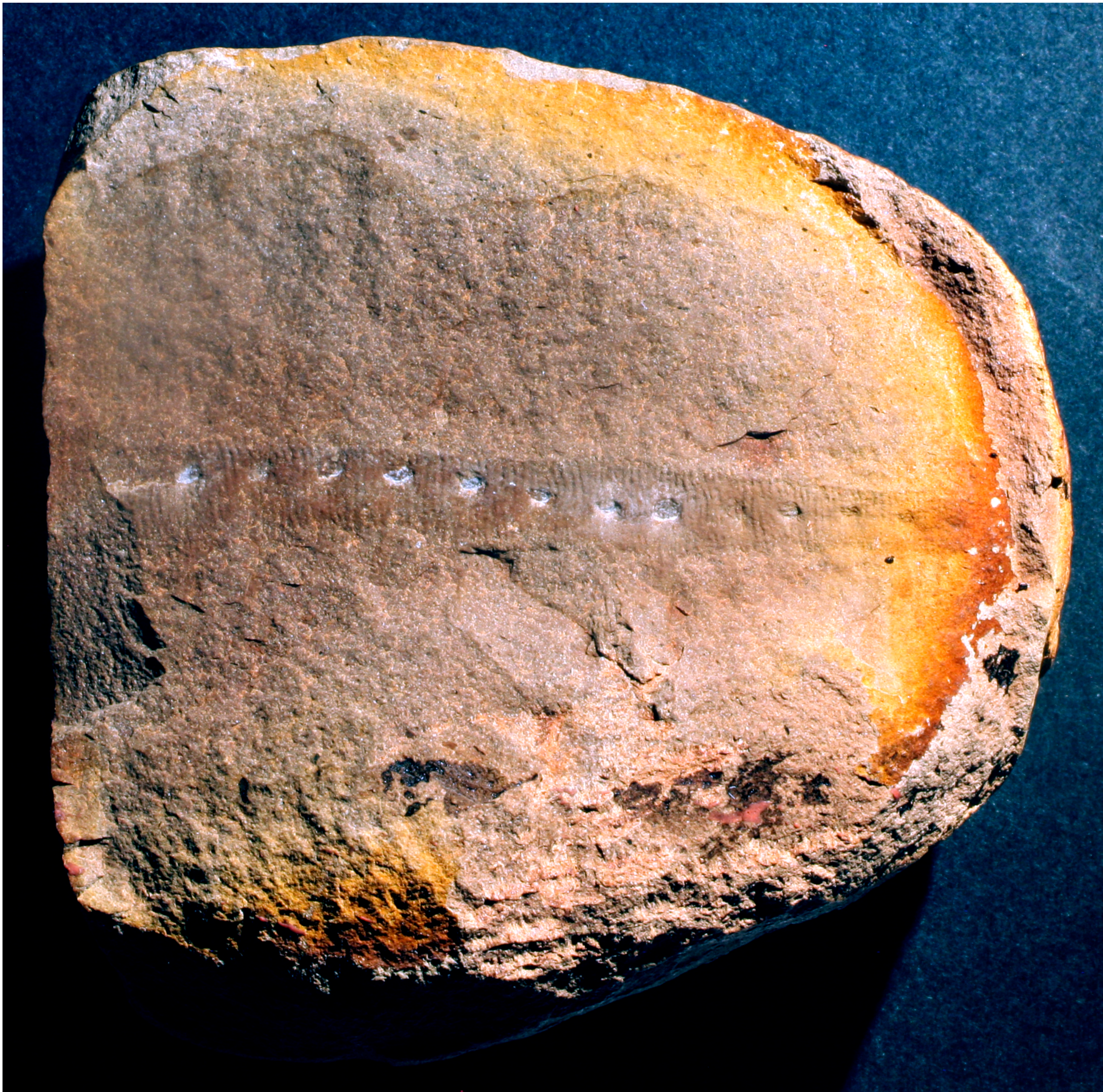

FIG 1C FULL

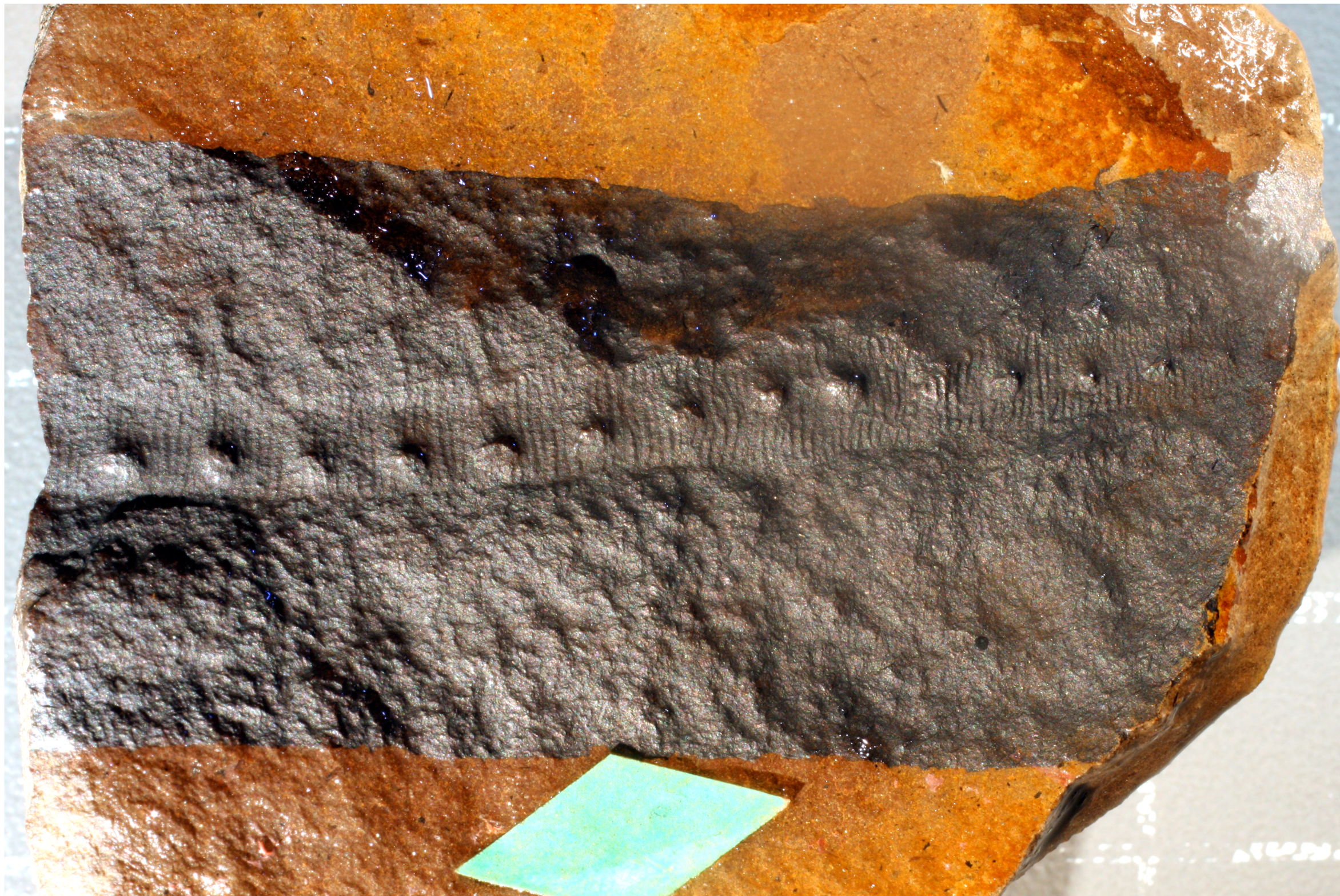

Supplement: Additional file 1: — Full resolution versions of images presented in Fig. 1a–d . RGB levels have been adjusted to better show the fossils, in some cases only in the region of the nodule that bears the fossil. For further details see caption at Fig. 1. (PDF 86713 kb) [file 12862_2016_582_MOESM1_ESM.pdf]

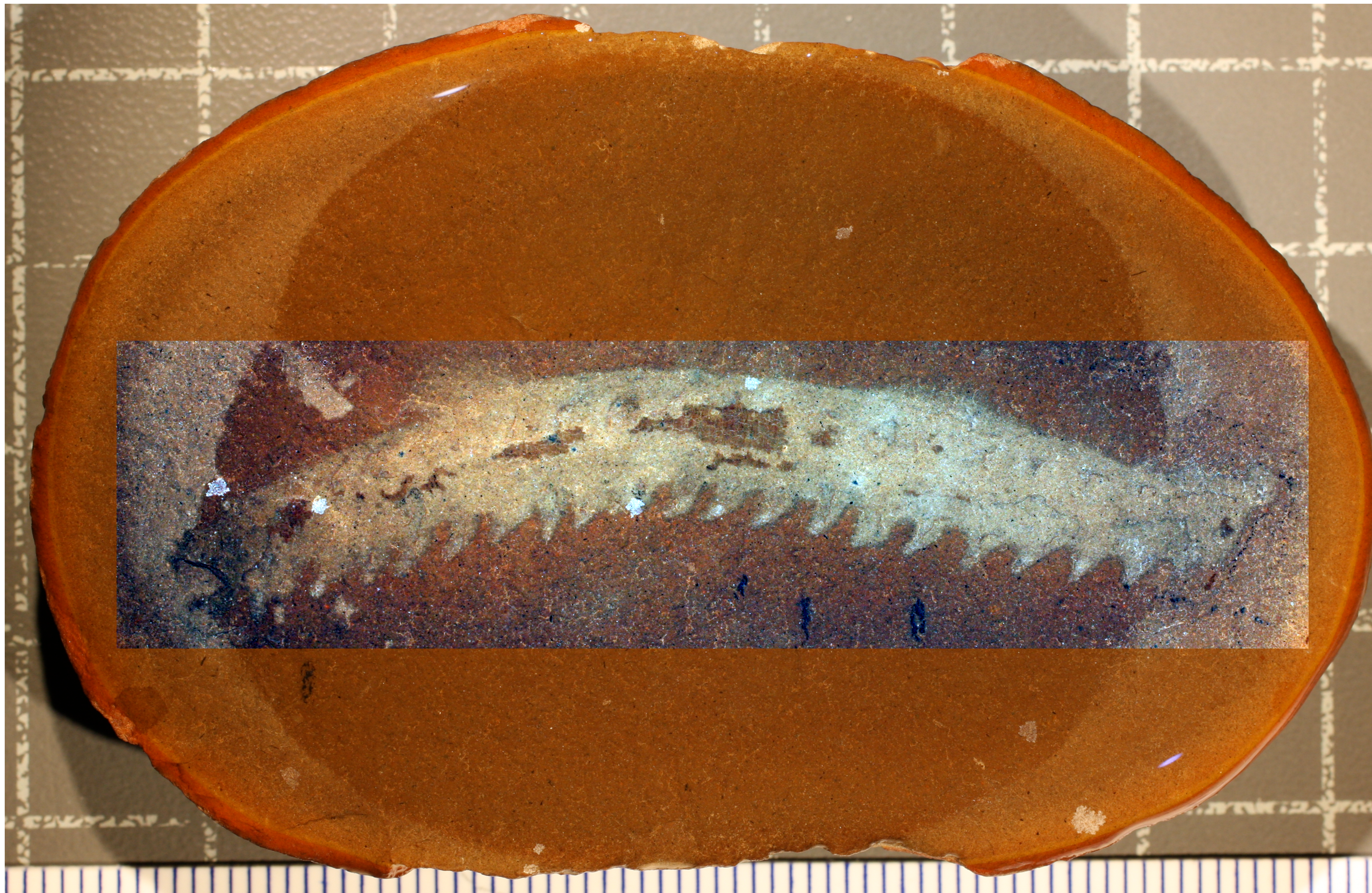

Fig 2A FULL

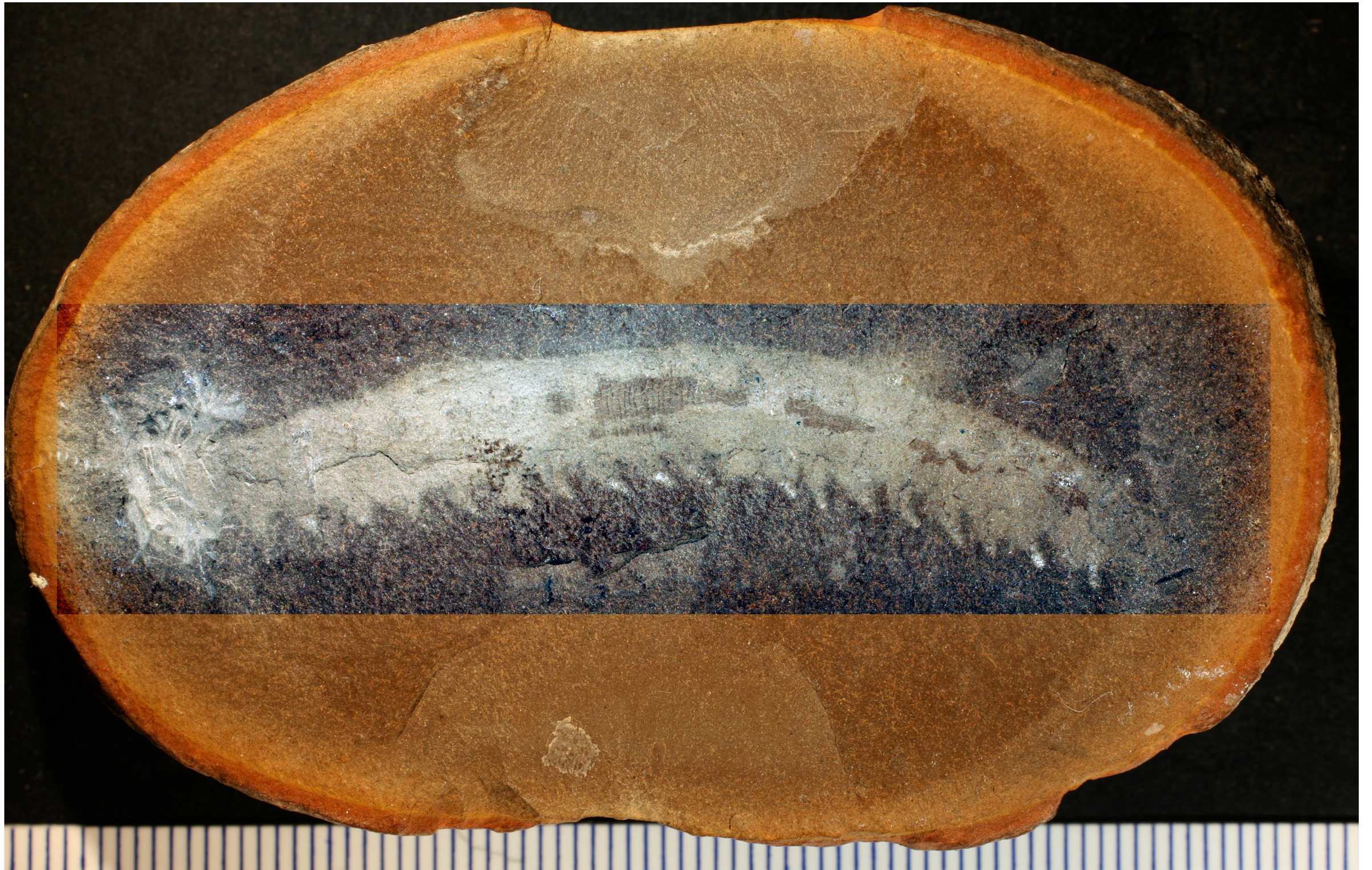

Fig 2C FULL

Supplement: Additional file 2: — Full resolution versions of images presented in Fig. 2a,c . RGB levels have been adjusted to better show the fossils, in some cases only in the region of the nodule that bears the fossil. For further details see caption at Fig. 2. (PDF 51834 kb) [file 12862_2016_582_MOESM2_ESM.pdf]

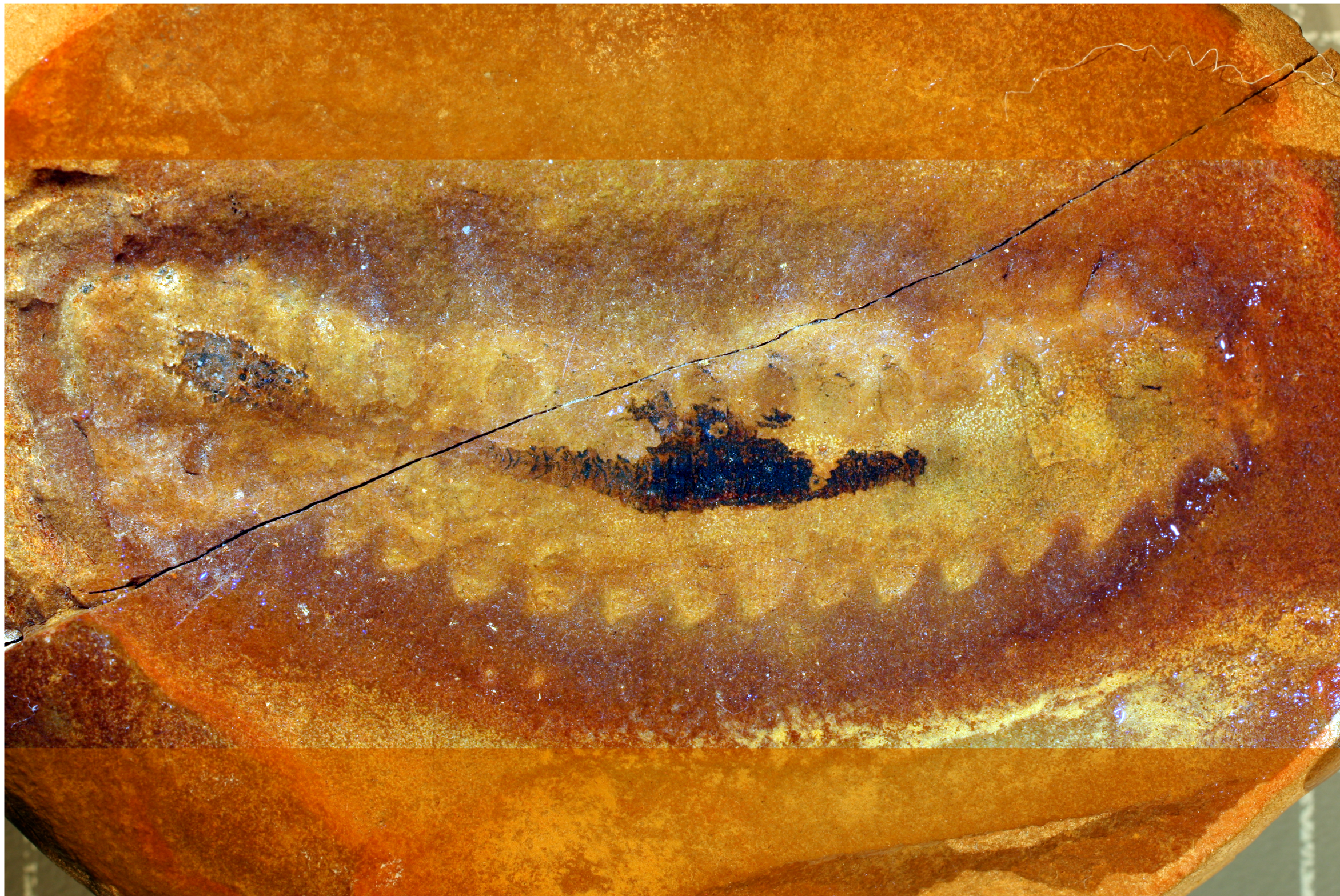

Fig 3A FULL

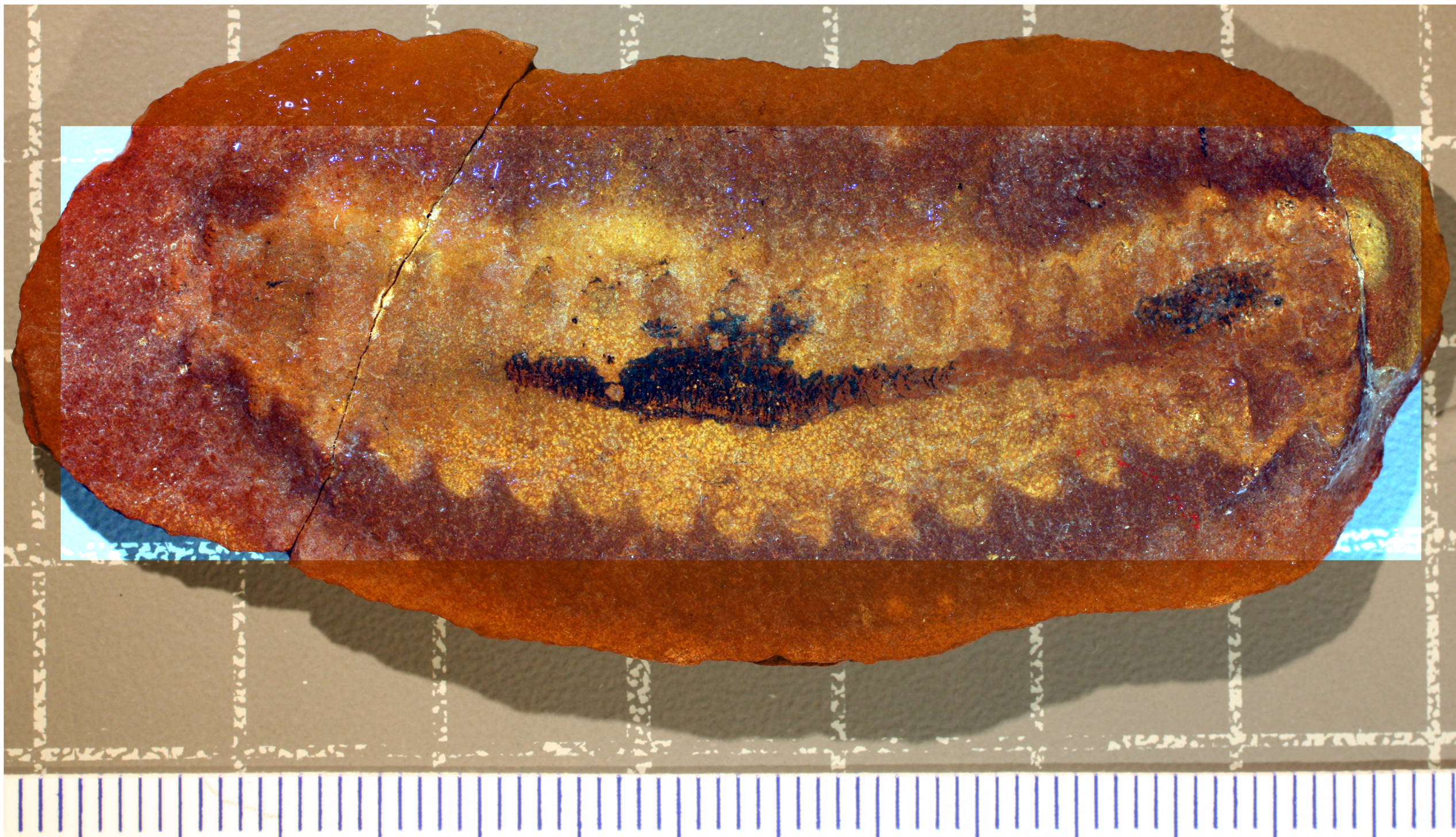

Fig 3C FULL

Supplement: Additional file 3: — Full resolution versions of images presented in Fig. 3a,c . RGB levels have been adjusted to better show the fossils, in some cases only in the region of the nodule that bears the fossil. For further details see caption at Fig. 3. (PDF 53604 kb) [file 12862_2016_582_MOESM3_ESM.pdf]

**Decay-informed coding for *Helenodora***

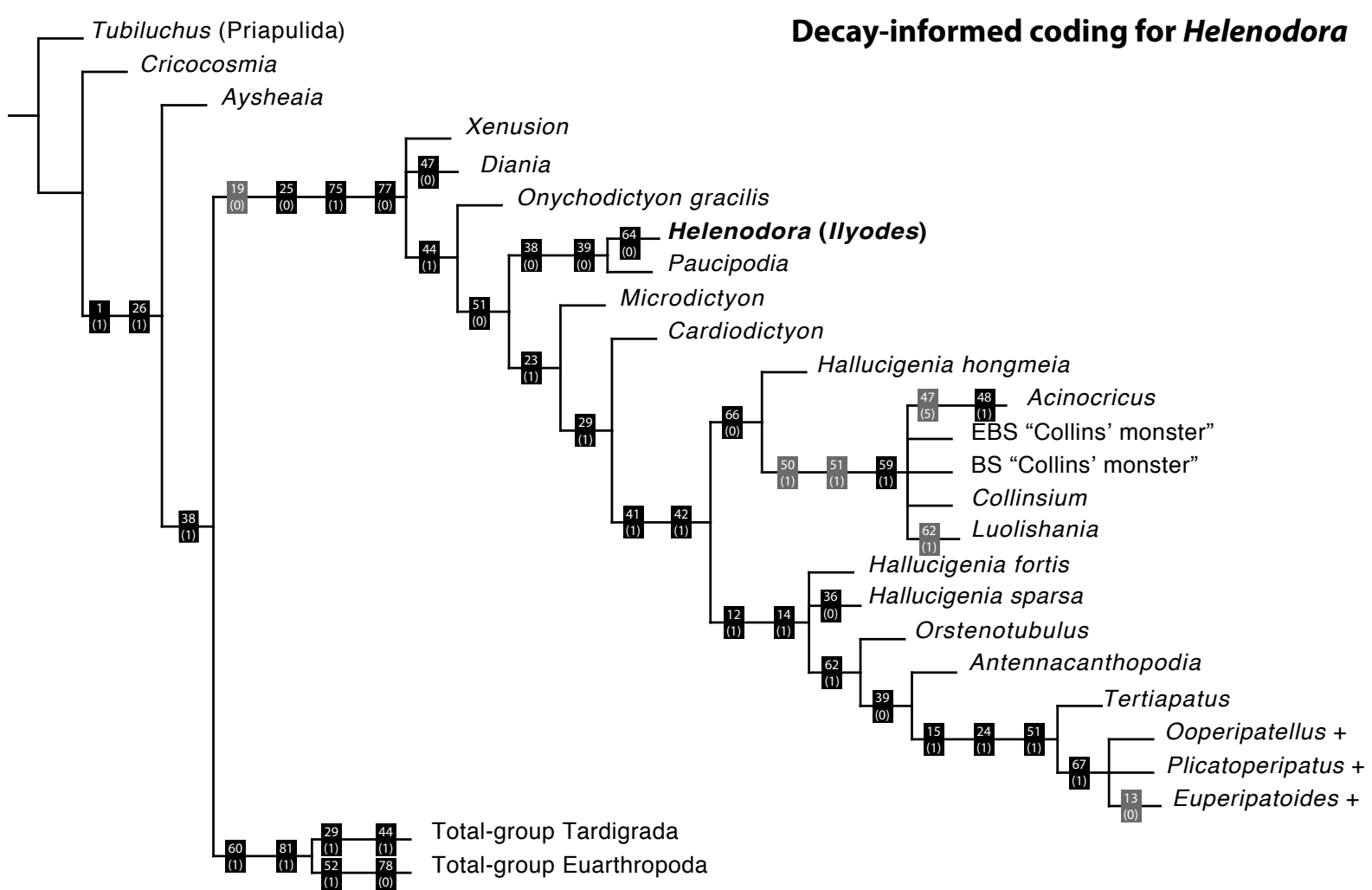

**Conservative coding for *Helenodora***

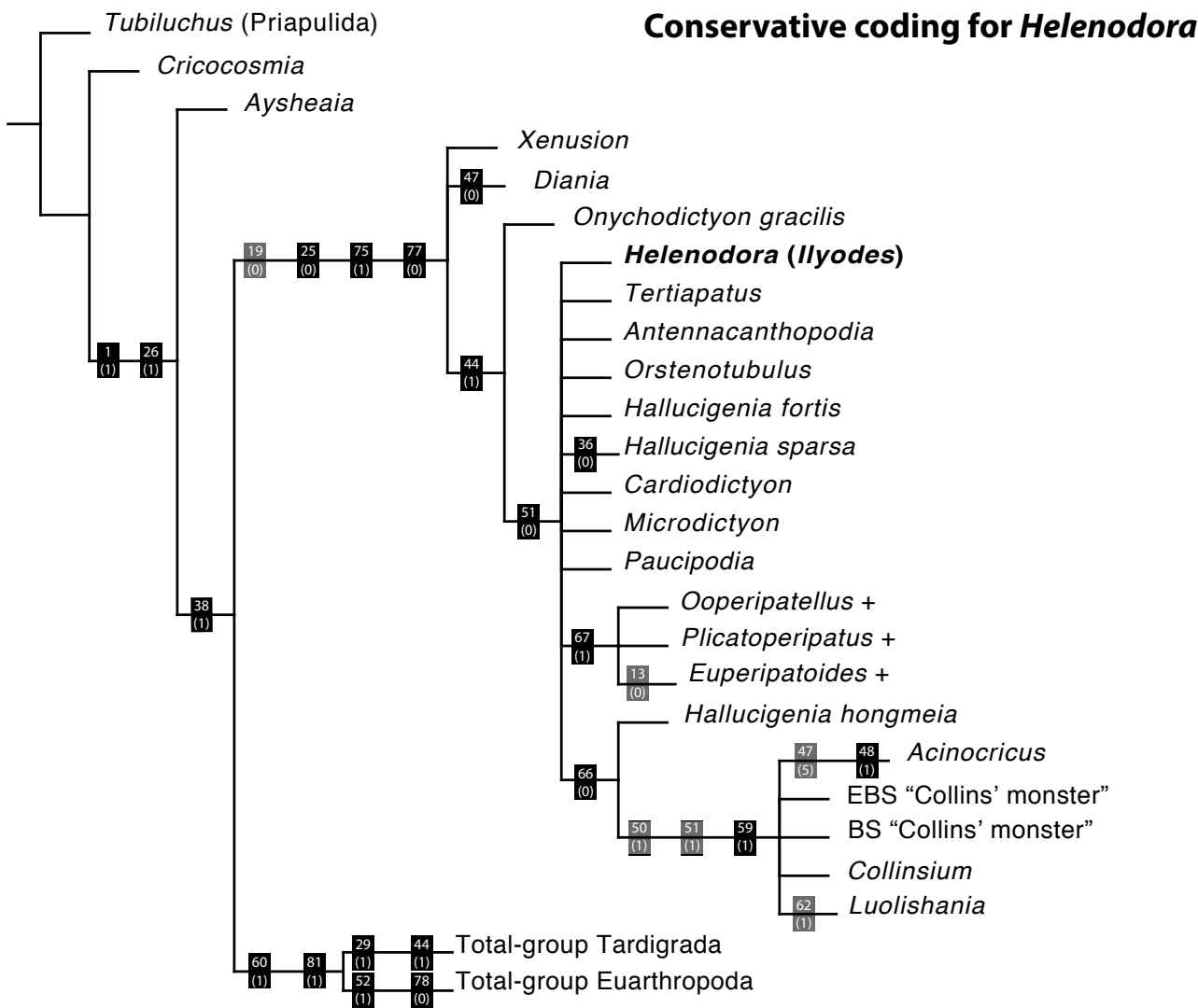

Supplement: Additional file 5: — Amended panarthropod phylogeny with synapomorphies mapped onto consensus tree topology. Upper topology derived from modifying the character coding for Helenodora inopinata (=Ilyodes) of Yang et al. [4] to reflect the new observations herein. Lower topology derived in the same way but with characters whose absence is inferred from taphonomic analysis coded as ambiguous. For details of how trees were generated see Methods and caption of fig. 8. Upper numbers refer to character number and lower (bracketed) numbers refer to character state, both from analysis of Yang et al. [4]. Grey boxes indicate synapomorphies not present in all trees used to draw the consensus. (PDF 369 kb) [file 12862_2016_582_MOESM5_ESM.pdf]
