# Supplementary material for: The impact of taphonomic data on phylogenetic resolution: Helenodora inopinata (Carboniferous, Mazon Creek Lagerstätte) and the onychophoran stem lineage
Source: BMC Evol Biol. 2016 Jan 22;16:19. doi: 10.1186/s12862-016-0582-7 (PMC4722706; doi:10.1186/s12862-016-0582-7)
Supplement: Additional file 6: — Character coding. Character descriptions and character states from Yang et al. [4], for those characters modified in this analysis (Table 1, Additional file 7), and/or those characters mapped as synapomophies (Additional file 5). (PDF 54 kb) [file 12862_2016_582_MOESM6_ESM.pdf]

Additional file 8: Character coding

Selected characters and character states from Yang et al. [1], for those referred to in 'The impact of taphonomic data on phylogenetic resolution: *Helenodora inopinata* (Carboniferous, Mazon Creek Lagerstätte) and the onychophoran stem lineage'.

1. Paired appendages
(0) absent
(1) present

12. Nature of first post-ocular (deutocerebral) appendage
(0) lobopodous ambulatory limb
(1) lobopodous sensorial limb
(2) loboodous limb with sclerotized jaw
(3) arthropodized antenniform with distinct podomeres
(4) arthropodized short great-appendage
(–) inapplicable: paired appendages (Character 1) absent

13. Inner blade of deutocerebral jaw with diastema
(0) absent
(1) present
(–) inapplicable: deutocerebral jaw (Character 12) absent

14. Deutocerebral limb pair structurally differentiated from rest of trunk appendages
(0) undifferentiated, or reduced in size only
(1) structurally differentiated
(–) inapplicable: paired appendages (Character 1) absent

15. Nature of second post-ocular (tritocerebral) appendage
(0) undifferentiated lobopodous limb
(1) specialized papillae
(2) arthropodized ambulatory limb with distinct podomeres
(3) arthropodized specialized post-antennal appendage
(-) inapplicable: paired appendages (Character 1) absent

19. Spines/spinules on pre-ocular (protocerebral) appendage
(0) absent
(1) present (radiodontans, gilled lobopodians, certain lobopodians)
(–) inapplicable: paired appendages (Character 1) absent; protocerebral appendages fused into labrum (Character 18)

23. Mouth opening orientation
(0) anterior
(1) ventral
(2) posterior

24. One or more pairs of appendages located anteriorly relative to the mouth opening
(0) absent
(1) present
(-) innaplicable; paired appendages (Character 1) absent

25. Radially symmetrical circumoral structures
(0) absent
(1) present

26. Nature of radial circumoral structures
(0) scalids
(1) oral papillae or lamellae
(2) radial plates organized as a mouth apparatus
(–) inapplicable: radial circumoral structures (Character 25) absent

29. Eyes
(0) absent
(1) present

36. Annulations
(0) absent
(1) present
(–) inapplicable: sclerotized dorsal integument with arthrodial membranes (Character 33) present

38. Organization of trunk annulation
(0) homonomous
(1) heteronomous
(–) inapplicable: annulations (Character 36) absent

39. Metamerically arranged dorsolateral epidermal specializations
(0) absent
(1) present

41. Proportions of epidermal trunk evaginations
(0) wider than tall (e.g. nodes or plates)
(1) taller than wide (e.g. spines)
(–) inapplicable: epidermal evaginations (Character 40) absent

42. Trunk epidermal evaginations with acute distal termination
(0) absent
(1) present
(–) inapplicable: epidermal evaginations (Character 40) absent

44. Sclerotization of epidermal evaginations
(0) absent
(1) present
(–) inapplicable: epidermal evaginations (Character 40) absent

46. Sclerites consist of a stack of constituent elements
(0) absent
(1) present
(–) inapplicable: sclerotized epidermal evaginations (Character 44) absent; terminal claws on limbs (Character 64) absent

47. Maximum number of primary dorsal epidermal specializations above each leg pair
(0) one
(1) two
(2) three
(3) four
(4) five
(5) seven
(–) inapplicable: epidermal specializations (Character 39) absent

48. 'Secondary' sclerotized dorsolateral spines
(0) absent
(1) present
(–) inapplicable: epidermal specializations (Character 39) absent; epidermal evaginations (Character 40) absent

50. Spacing between dorsolateral epidermal specializations along longitudinal body axis
(0) epidermal specializations regularly spaced
(1) epidermal specializations irregularly spaced
(–) inapplicable: epidermal specializations (Character 39) absent

51. Papillae on trunk annulations
(0) absent
(1) present
(–) inapplicable: annulations (Character 36) absent

52. Serially repeated mid-gut glands
(0) absent
(1) reniform, submillimetric lamellar

59. Secondary structures on lobopodous limbs
(0) absent
(1) present
(–) inapplicable: limbs (Character 8) not lobopodous

60. Nature of secondary structures
(0) spines/setae
(1) appendicules
(–) inapplicable: secondary structures on the lobopodous limbs (Character 59) absent

62. Papillae on lobopodous limbs
(0) absent
(1) present
(–) inapplicable: limbs (Character 8) are not lobopodous

64. Terminal claws on trunk limbs
(0) absent
(1) present
(–) inapplicable: paired appendages (Character 1) absent

65. Terminal claws with multiple branches
(0) absent
(1) present
(–) inapplicable: terminal claws (Character 64) absent

66. Number of claws on trunk limbs
(0) one
(1) two
(2) three
(3) four
(4) seven
(–) inapplicable: terminal claws (Character 64) absent

67. Differentiated distal foot in lobopodous trunk limbs
(0) absent
(1) present
(–) inapplicable: paired appendages (Character 1) absent; post-ocular appendages (Character 8); inner branch modified as lateral flaps (Character 11)

75. Limbless posterior extension of the lobopodous trunk beyond last appendage pair
(0) absent
(1) present
(–) inapplicable: paired appendages (Character 1) absent; dorsal trunk covered by sclerotized plates (Character 33) present

77. Posteriormost trunk appendage pair structurally differentiated
(0) undifferentiated
(1) differentiated
(–) inapplicable: paired appendages (Character 1) absent

80. Claws on posterior appendages directed anteriad
(0) normal orientation (claws pointing posteriad)
(1) rotated anteriad
(–) inapplicable: appendages lack terminal claws (Character 64); appendicular tail (Character 78) present

1. Yang J, Ortega-Hernández J, Gerber S, Butterfield NJ, Hou J, Lan T, Zhang X: **A superarmored lobopodian from the Cambrian of China and early disparity in the evolution of Onychophora**. *Proc Natl Acad Sci* 2015:201505596.
