# Supplementary material for: The impact of taphonomic data on phylogenetic resolution: Helenodora inopinata (Carboniferous, Mazon Creek Lagerstätte) and the onychophoran stem lineage
Source: BMC Evol Biol. 2016 Jan 22;16:19. doi: 10.1186/s12862-016-0582-7 (PMC4722706; doi:10.1186/s12862-016-0582-7)
Supplement: Additional file 7: — Full details of modifications to the character coding for Helenodora inopinata (= Ilyodes ). Based on data presented by Yang et al. [4]. Changes highlighted in bold. (PDF 45 kb) [file 12862_2016_582_MOESM7_ESM.pdf]

**Character from Yang et al. 2015**	**Original coding**	**Our decay-informed coding**	**Our conservative coding**	**Notes**
12. Nature of first post-ocular (deutocerebral) appendage	(?) ambiguous	**(0) lobopodous ambulatory limb**	(?) ambiguous	Taphonomic analysis indicates lack of jaws is real, not result of decay
13. Inner blade of deutocerebral jaw with diastema	(?) ambiguous	**(-) inapplicable: deutocerebral jaw (Character 12) absent**	(?) ambiguous	Taphonomic analysis indicates lack of jaws is real, not result of decay
14. Deutocerebral limb pair structurally differentiated from rest of trunk appendages	(?) ambiguous	**(0) undifferentiated, or reduced in size only**	(?) ambiguous	Taphonomic analysis indicates lack of jaws is real, not result of decay
15. Nature of second post-ocular (tritocerebral) appendage	(1) specialized papillae	**(0) undifferentiated lobopodous limb**	**(?) ambiguous**	*Helenodora* lacks slime papillae
24. One or more pairs of appendages located anteriorly relative to the mouth opening	(1) present	**(?) ambiguous**	**(?) ambiguous**	*Helenodora* does not preserve a mouth, so this position is ambiguous
36. Annulations	(?) ambiguous	**(1) present**	**(1) present**	Present in new material
38. Organization of trunk annulation	(?) ambiguous	**(0) homonomous**	**(0) homonomous**	Present in new material
46. Sclerites consist of a stack of constituent elements	(?) ambiguous	**(–) inapplicable: terminal claws on limbs (Character 64) absent**	(?) ambiguous	Taphonomic analysis indicates lack of claws is real, not result of decay
52. Serially repeated mid-gut glands	(0) absent	**(?) ambiguous**	**(?) ambiguous**	No internal anatomy is preserved
62. Papillae on lobopodous limbs	(1) present	**(?) ambiguous**	**(?) ambiguous**	Features describe as papillae are treated here as equivocal
64. Terminal claws on trunk limbs	(1) present	**(0) absent**	**(?) ambiguous**	Taphonomic analysis indicates lack of claws is real, not result of decay
65. Terminal claws with multiple branches	(0) absent	**(–) inapplicable: terminal claws (Character 64) absent**	**(?) ambiguous**	Taphonomic analysis indicates lack of claws is real, not result of decay
66. Number of claws on trunk limbs (0)	(1) two	**(–) inapplicable: terminal claws (Character 64) absent**	**(?) ambiguous**	Taphonomic analysis indicates lack of claws is real, not result of decay
67. Differentiated distal foot in lobopodous trunk limbs	(?) ambiguous	**(0) absent**	**(0) absent**	Limb morphology clear in new material
75. Limbless posterior extension of the lobopodous trunk beyond last appendage pair	(?) ambiguous	**(1) present**	**(1) present**	Posterior morphology clear in new material
80. Claws on posterior appendages directed anteriad	(?) ambiguous	**(–) inapplicable: terminal claws (Character 64) absent**	(?) ambiguous	Taphonomic analysis indicates lack of claws is real, not result of decay
