# Supplementary material for: The impact of taphonomic data on phylogenetic resolution: Helenodora inopinata (Carboniferous, Mazon Creek Lagerstätte) and the onychophoran stem lineage
Source: BMC Evol Biol. 2016 Jan 22;16:19. doi: 10.1186/s12862-016-0582-7 (PMC4722706; doi:10.1186/s12862-016-0582-7)
Supplement: Additional file 4: — Full resolution versions of images presented in Fig. 4a–h . RGB levels have been adjusted to better show the fossils, in some cases only in the region of the nodule that bears the fossil. For further details see caption at Fig. 4. (PDF 219449 kb) [file 12862_2016_582_MOESM4_ESM.pdf]

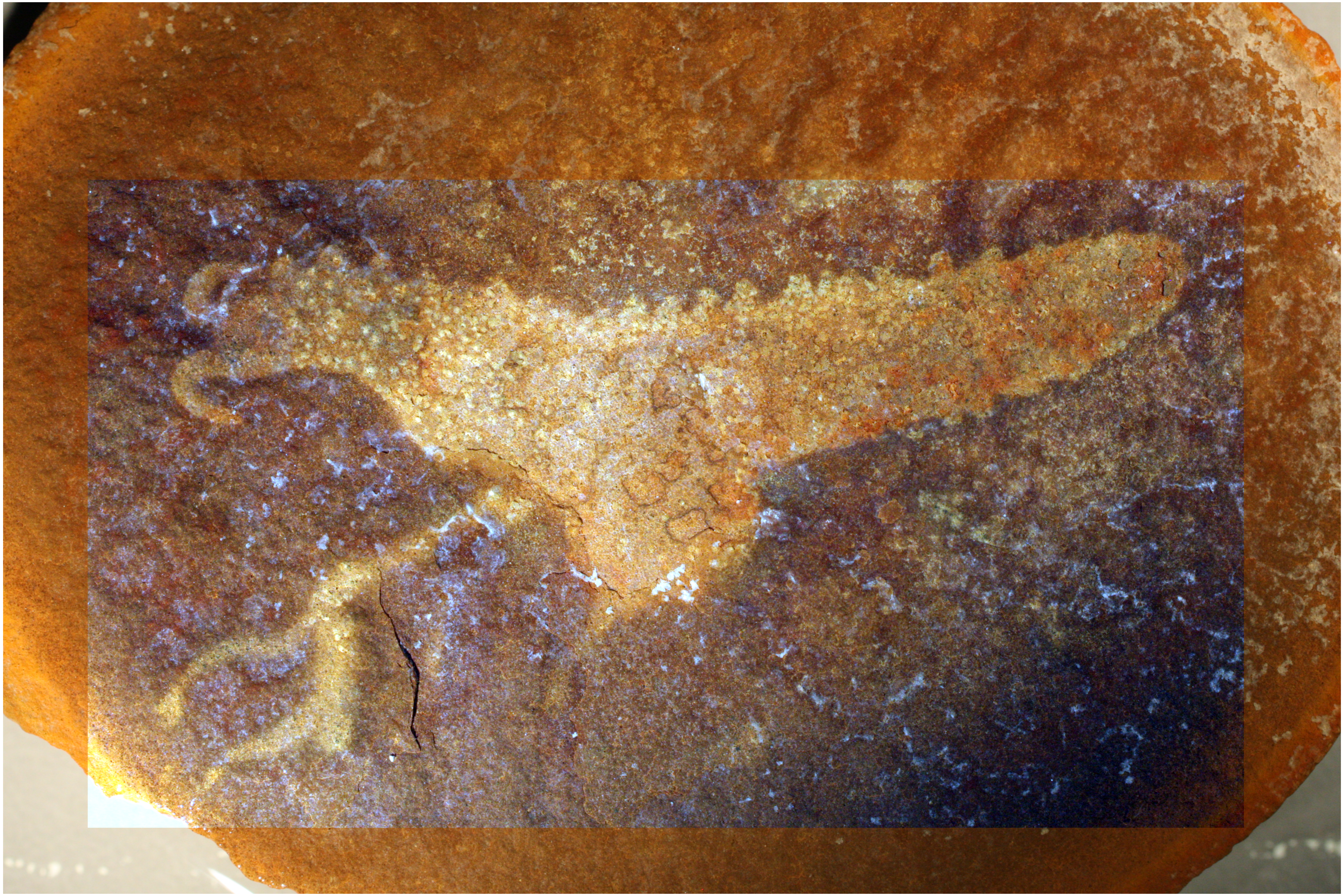

Fig 4A FULL

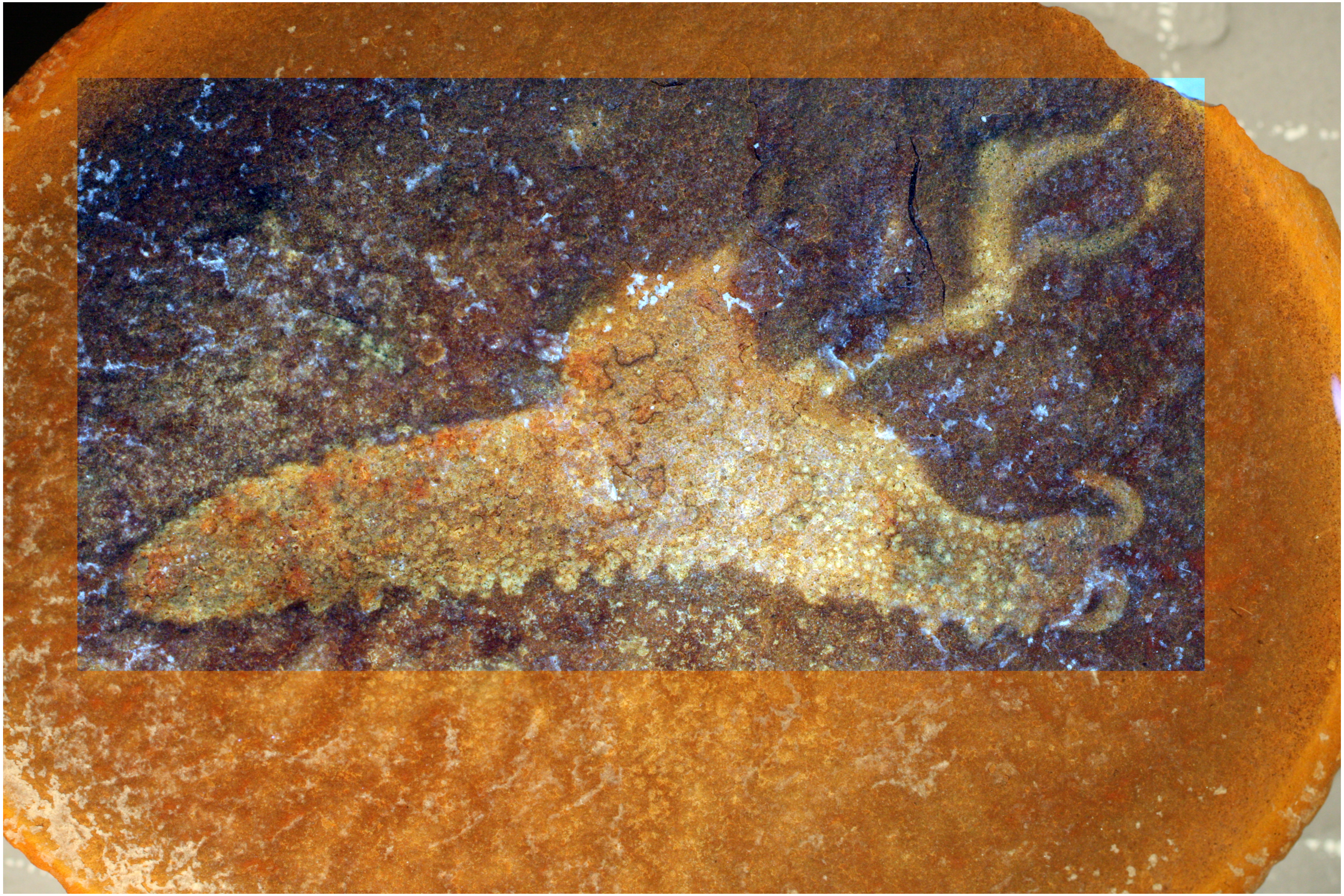

FIG 48 FULL

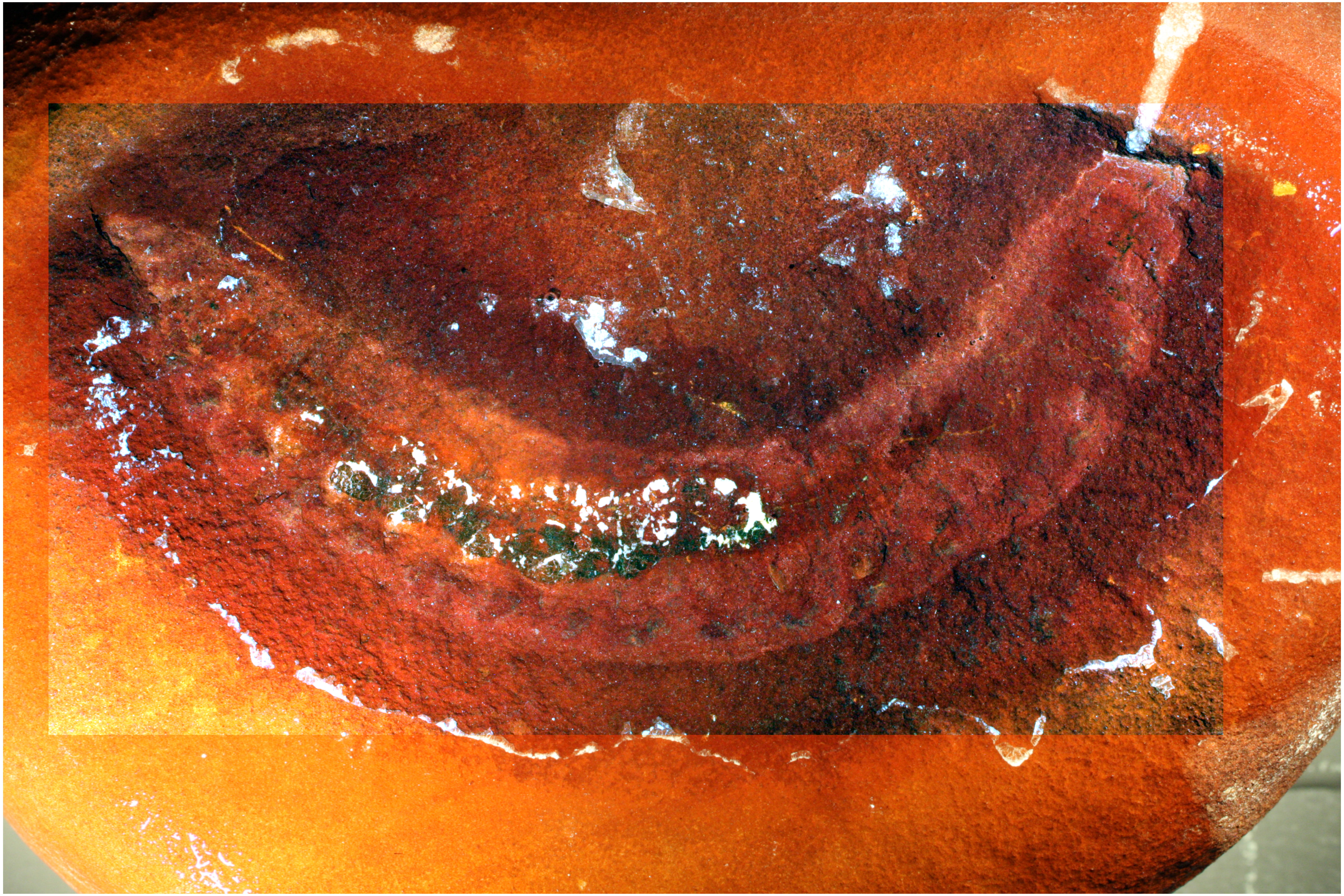

FIG 4C FULL

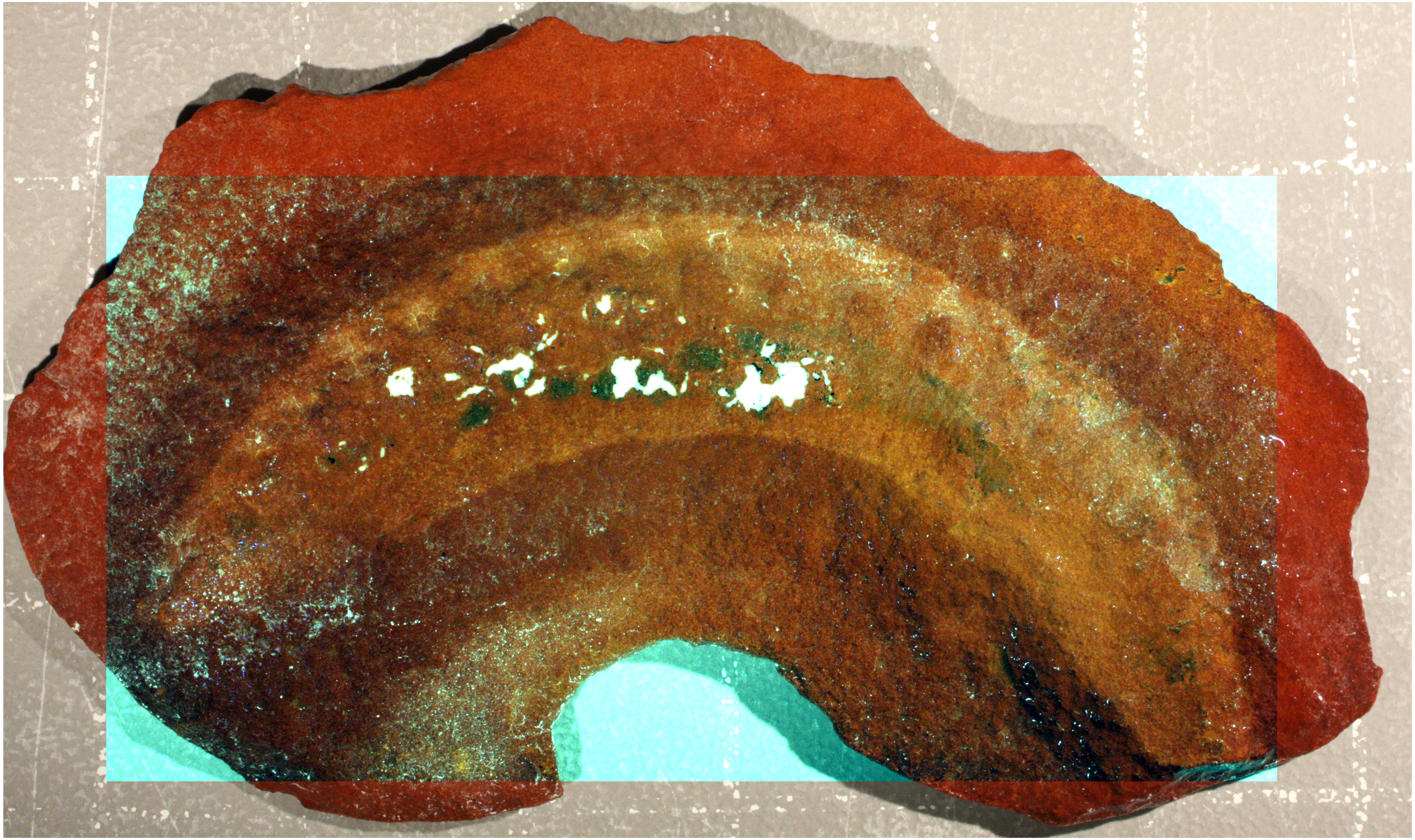

Fig 4D FULL

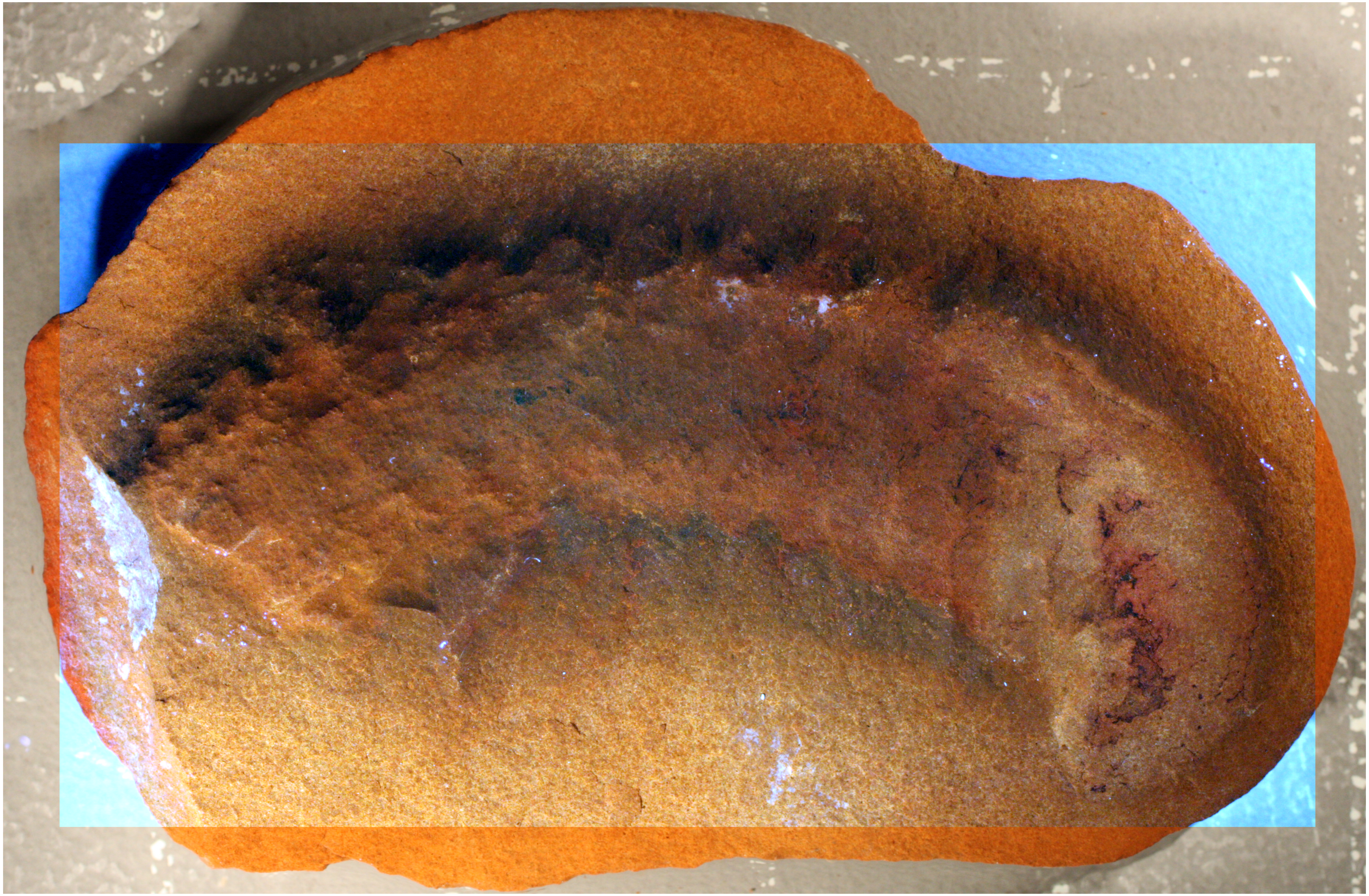

Fig 4E FULL

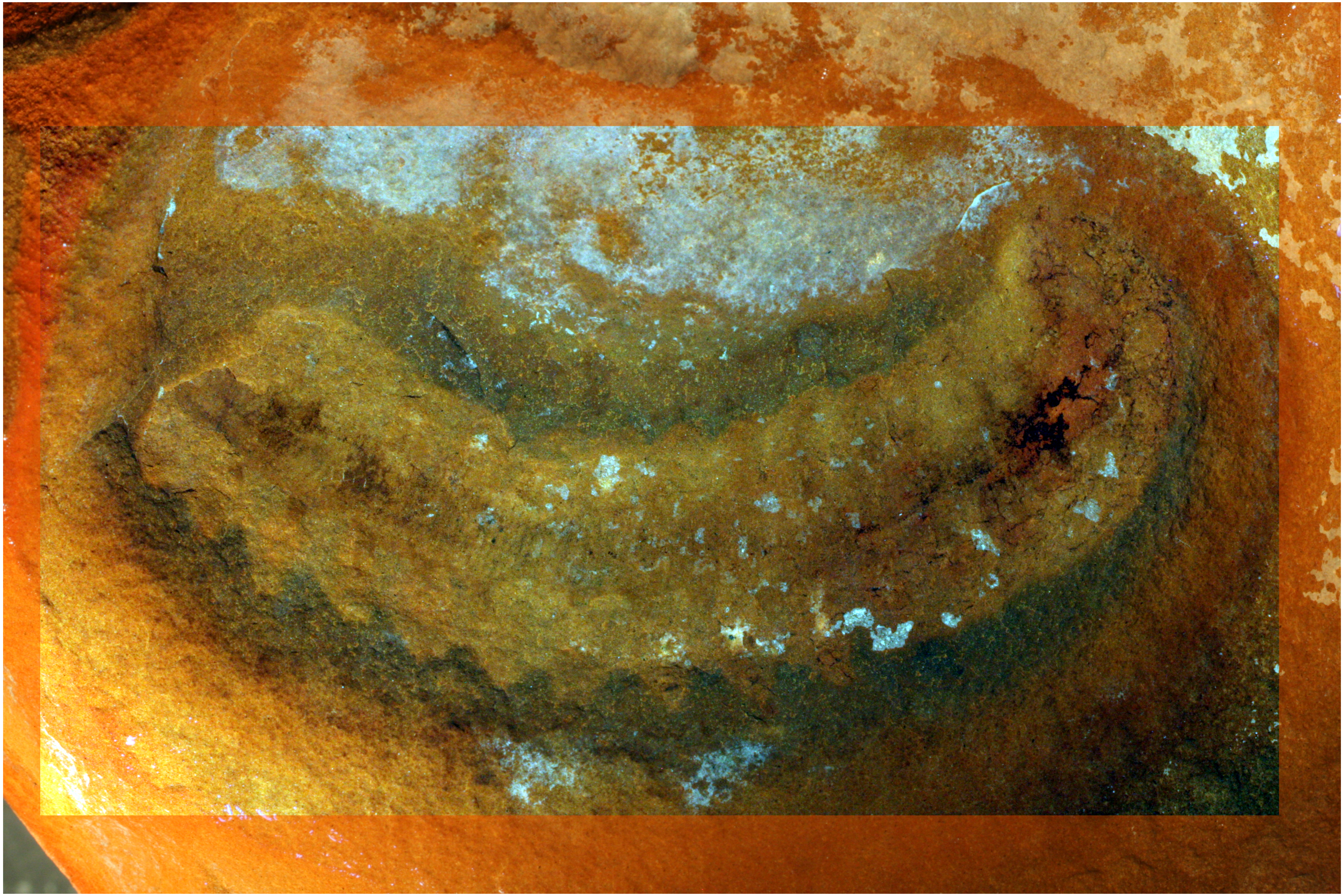

Fig 4F FULL

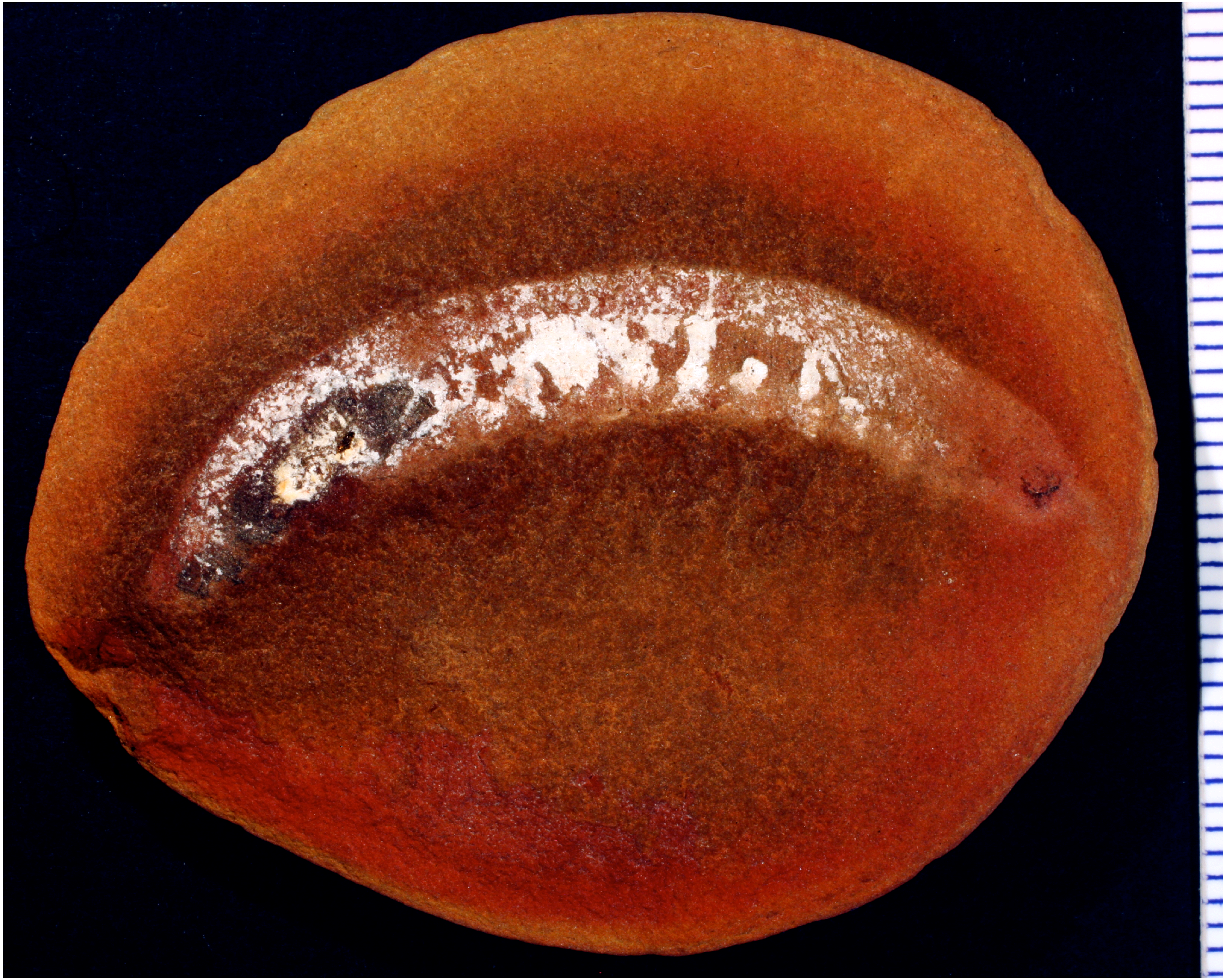

Fig 4G FULL

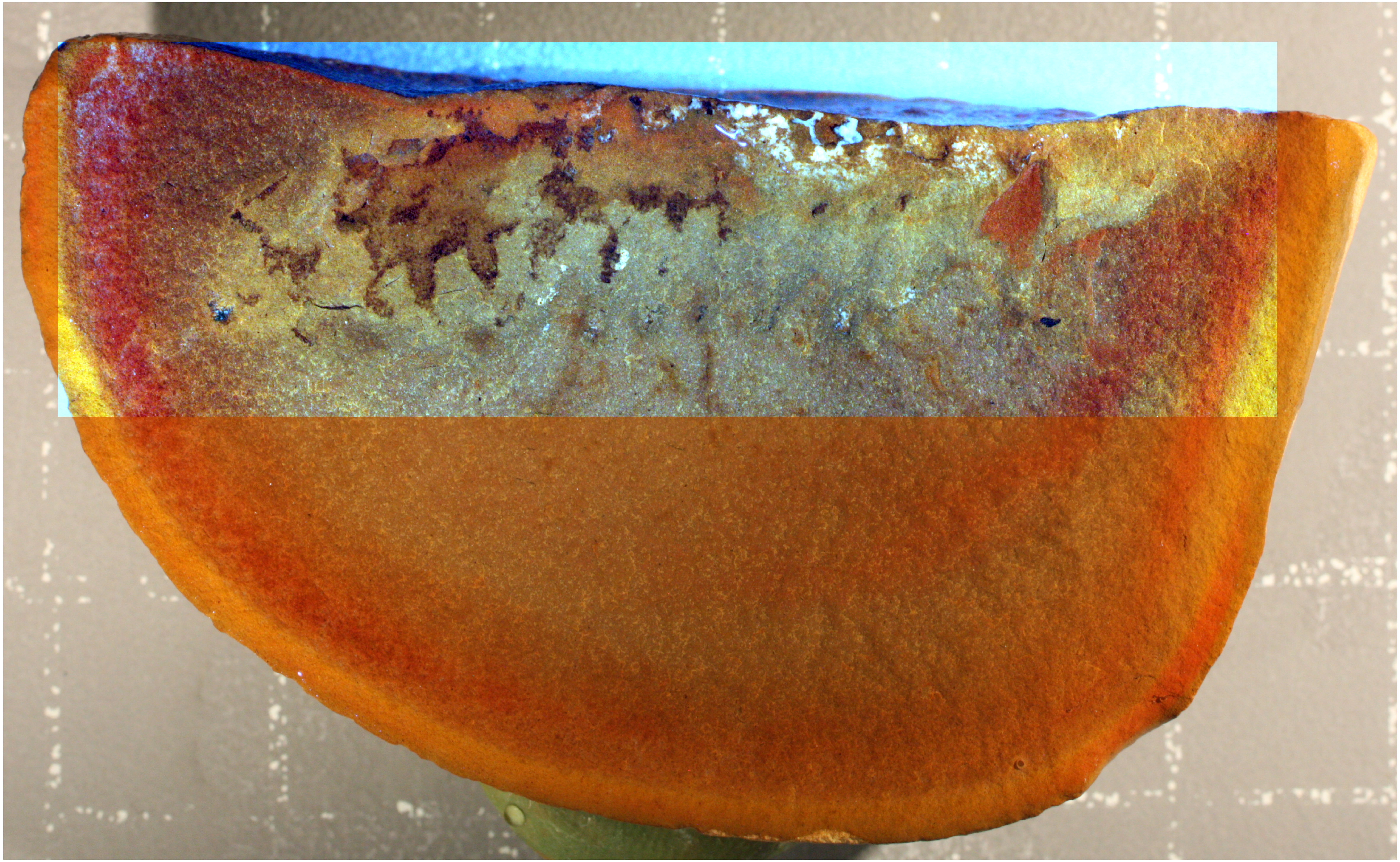

Fig 4H FULL
